# Efficacy of botanical extracts for knee osteoarthritis: a network meta-analysis of randomized controlled trials

**DOI:** 10.3389/fphar.2025.1619589

**Published:** 2025-10-07

**Authors:** Chao Yan, Xiaotao Du, Yan Liu, Fan Xia, Hongling Yu, Yongliang Zhu, Tianyi Bao

**Affiliations:** ^1^ Department of Orthopaedics, Nanjing Central Hospital, Nanjing, China; ^2^ Nanjing Drum Tower Hospital, The Affiliated Hospital of Nanjing University Medical School, Nanjing, China

**Keywords:** botanical extracts, knee osteoarthritis, system review, network, meta

## Abstract

**Background:**

Traditional botanical drugs and medicinal plants, along with their metabolite extracts, have exhibited considerable potential in the management of knee osteoarthritis (KOA) due to their natural properties, favorable safety profiles, and minimal adverse effects.

**Objective:**

This study aimed to evaluate the therapeutic efficacy of various botanical and medicinal plant extracts on KOA. Search Methods: We conducted a comprehensive literature search across PubMed, Embase, Cochrane Library, and Web of Science, focusing exclusively on randomized controlled trials (RCTs) that investigated the efficacy of botanical and medicinal plant extracts for KOA. Selection Criteria: Studies were included if they met the following criteria: (1) experimental groups receiving single botanical drugs or plant extracts for KOA; (2) control groups comprising patients receiving placebo or standard care; (3) clinical RCT designs; and (4) outcome measures including at least one of the following: Western Ontario and McMaster Universities Osteoarthritis Index (WOMAC), Visual Analogue Scale (VAS), Short Form 36 Health Survey (SF-36), Knee injury and Osteoarthritis Outcome Score (KOOS), Lequesne’s Pain-Function Index (LPFI), Japanese Orthopaedic Association Score (JOA). Data Collection and Analysis: The methodological quality of the included studies was assessed using the Cochrane risk of bias tool, and data analysis was performed using appropriate statistical software.

**Results:**

A total of 36 RCTs, encompassing 3,285 participants, were included in this review. Network meta-analysis revealed that compared to the placebo control group, Cucumis sativus (CS) extract [MD = 6.65, 95% CI = (3.83, 9.48)] significantly improved pain scores; Ashwagandha extract [MD = 4.16, 95% CI = (2.43, 5.90)] was more effective in reducing stiffness scores; and CS extract [MD = 4.28, 95% CI = (2.08, 6.49)] significantly improved function scores.

**Conclusion:**

Based on Ranking Plot of the Network, we can state that CS extract is recommended as the most effective botanical and medicinal plant extract for KOA treatment. However, further studies are required to draw definitive conclusions. Given that there are only two studies with high homogeneity but small sample size for CS extract, the first result should be regarded as an exploratory signal and needs to be verified by a large sample multi-center RCT with independent teams.

**Clinical Trial Registration:**

https://www.crd.york.ac.uk/prospero/display_record.php?ID=CRD42024617459, identifier CRD42024617459.

## 1 Introduction

Knee Osteoarthritis (KOA) is a degenerative disease primarily affecting the knee joint function of middle-aged and elderly populations (Giordano et al., 2020). Its pathological characteristics include joint space narrowing, subchondral bone sclerosis, and osteophyte formation, with clinical symptoms such as joint pain, restricted mobility, crepitus during movement, joint deformity, and muscle atrophy (Sharma, 2021). Remarkably, KOA has contributed more to the global burden of OA than any other site (Long et al., 2022). With the global trend of population aging, the prevalence of KOA continues to rise, becoming an increasingly serious public health issue worldwide (Menon and Mishra, 2018) and placing substantial pressure on healthcare systems and economies (Bedenbaugh et al., 2021; Jin et al., 2023). Data released by the World Health Organization in February 2021 reported that the number of osteoarthritis patients worldwide had reached approximately 343 million. In China, the overall prevalence of KOA is 8.1%, with rates of 5.2% in individuals aged 50 and above and rising to 11% in those aged 60 and older (Chen et al., 2022). By comparison, the overall prevalence rates of KOA in the United States and Europe are 4.3% and 5.3% (Chen et al., 2022), respectively, further highlighting the extensive impact of this condition.

Currently, the treatment of KOA primarily includes non-pharmacological interventions, pharmacological therapies, and surgical procedures (Charlesworth et al., 2019). Non-pharmacological interventions encompass patient education, moderate physical activity, mobility support, and physical therapy, aiming to reduce joint load, improve joint function, and enhance quality of life. Pharmacological therapies include oral administration of chondroprotective agents such as glucosamine and chondroitin sulfate, as well as analgesics like nonsteroidal anti-inflammatory drugs (NSAIDs) to alleviate pain and suppress inflammation (Kolasinski et al., 2020). Surgical interventions, such as joint replacement surgery, are typically recommended for patients with more severe conditions (Hannon et al., 2023). However, while conventional treatments can alleviate the symptoms of KOA to some extent, they cannot achieve a complete cure. Moreover, long-term use of certain medications may result in adverse effects, such as gastrointestinal discomfort and liver or kidney damage (da Costa et al., 2017). Therefore, the search for safer and more effective treatment options has become a major focus of current research efforts.

As there are currently no specific drugs for the treatment of KOA, traditional botanical drugs or medicinal plants and their extracted metabolites have shown significant potential due to their natural origins, high safety profile, and minimal side effects. The screening of botanical drugs and elucidation of their mechanisms of action are of great importance in the treatment of KOA (Wang et al., 2022). Many botanical and medicinal plant extracts, such as curcumin, ginger extract, and willow bark extract, have been found to possess anti-inflammatory, anti-swelling, and analgesic properties, effectively alleviating the symptoms of KOA. Numerous botanical drugs and their secondary metabolites, including extracts from Scutellaria baicalensis, turmeric, chicory root, frankincense, and ginger, have demonstrated potential therapeutic activity against KOA (Wang et al., 2022). The underlying mechanisms include reducing oxidative stress, inhibiting chondrocyte apoptosis, promoting autophagy, and modulating the NF-κB signaling pathway (Ziadlou et al., 2019). In recent years, significant progress has been made in the application of botanical drugs for the treatment of KOA (Walzer et al., 2015). Multiple clinical trials have indicated that botanical drugs or their active metabolites play an important role in alleviating KOA symptoms. Meta-analyses have shown that botanical medicines exhibit better efficacy in treating KOA compared to placebos and some conventional biomedicines (Lin et al., 2022). Additionally, combining traditional Chinese medicine with Biomedicine has proven more effective than using biomedicine alone (Liang et al., 2022). However, due to potential biases in the included studies, further research is needed to identify the active metabolites of botanical medicines and clarify their mechanisms of action.

Network meta-analysis (NMA) is an evidence-based technique used to compare the effectiveness of multiple interventions for a specific disease and to rank these treatments based on their efficacy (Rouse et al., 2017). In this study, we employed a network meta-analysis to compare different botanical and medicinal plant extracts, evaluate their effectiveness in treating patients with KOA, and provide patients and clinicians with a clearer understanding of the therapeutic potential of these extracts. Our objective is to assess the effects of these botanical and medicinal plant extracts on KOA patients and provide evidence-based recommendations for both patients and clinicians.

## 2 Materials and methods

### 2.1 Search strategies

Researchers retrieved data from four electronic databases (PubMed, EMBASE, Cochrane Central Register of Controlled Trials, and Web of Science) spanning from their inception to October 2024. The search strategy was developed based on the PICOS framework: (P) Population: Knee Osteoarthritis; (I) Intervention: botanical and medicinal plant extracts; (C) Comparison: Placebo or conventional treatment measures; (O) Outcome: Functional scores of patients with knee osteoarthritis; (S) Study design: Randomized controlled trials (RCTs). The detailed search strategy is presented in Supplementary Table S5. The PICOTS framework is detailed in Supplementary Table S6.

### 2.2 Inclusion criteria

1. Experimental groups of various single botanical drugs or plant extracts for knee osteoarthritis included: *Zingiber officinale* Roscoe [Zingiberaceae; Zingiberis rhizoma] (Ginger), *Ananas comosus* (L.) Merr. [Bromeliaceae; Ananasis caulis] (Pineapple, bromelain), *Boswellia serrata* Roxb. ex Colebr. [Burseraceae; Olibanum indicum] (Indian frankincense, BS), *Passiflora edulis* Sims [Passifloraceae; Passiflorae edulis pericarpium] (Passion fruit peel, PFP), *Derris scandens* (Roxb.) Benth. [Fabaceae; Derridis scandentis caulis] (DSB), *Curcuma longa* L. [Zingiberaceae; Curcumae longae rhizoma] (Turmeric, Curcuma), *Sesamum indicum* L. [Pedaliaceae; Sesami semen] (Sesame), *Prunus cerasus* L. [Rosaceae; Pruni cerasi fructus] (Tart Cherry), *Olea europaea* L. [Oleaceae; Oleae folium] (Olive leaf, OL), *Punica granatum* L. [Lythraceae; Granati pericarpium] (Pomegranate), *Elaeagnus angustifolia* L. [Elaeagnaceae; Elaeagni angustifoliae fructus] (EA, Russian olive), *Withania somnifera* (L.) Dunal [Solanaceae; Withaniae radix] (Ashwagandha), *Argania spinosa* (L.) Skeels [Sapotaceae; Arganiae oleum] (Argan), *Camellia sinensis* (L.) Kuntze [Theaceae; Theae folium] (Green tea, GT), *Psidium guajava* L. [Myrtaceae; Psidii guajavae folium] (Guava leaf, GL), *Momordica charantia* L. [Cucurbitaceae; Momordicae fructus] (MC, bitter melon), *Cucumis sativus* L. [Cucurbitaceae; Cucumeris sativi fructus] (CS, cucumber), *Allium sativum* L. [Amaryllidaceae; Allii sativi bulbus] (Garlic), *Andrographis paniculata* (Burm.f.) Nees [Acanthaceae; Andrographidis herba] (AP), *Foeniculum vulgare* Mill. [Apiaceae; Foeniculi fructus] (Fennel), *Chrysanthemum zawadskii* var. latilobum [Asteraceae; Chrysanthemi flos] (CZ), *Rubus idaeus* L. [Rosaceae; Rubi idaei folium] (RIL, raspberry leaf), *Nigella sativa* L. [Ranunculaceae; Nigellae semen] (NS, black seed), *Perilla frutescens* (L.) Britton [Lamiaceae; Perillae folium] (Zisu), *Delphinium denudatum* Wall. ex Hook. f. and Thomson [Ranunculaceae; Delphinii radix] (Jadwar), and *Drimia maritima* (L.) Stearn [Asparagaceae; Scillae bulbus] (SO, Squill oxymel); All botanical drugs were selected and reported according to the guidelines of the “Consortium for Phytochemical Characterization of Medicinal Plants (ConPhyMP)”; (2) Control groups consisting of patients who received only placebo or usual treatment; (3) Study designs limited to clinical RCTs; and (4) Outcome measures that included at least one of the following: Western Ontario and McMaster Universities Osteoarthritis Index (WOMAC), Visual Analogue Scale (VAS), Short Form 36 Health Survey (SF-36), Knee injury and Osteoarthritis Outcome Score (KOOS), Lequesne’s Pain-Function Index (LPFI), Japanese Orthopaedic Association Score (JOA).

### 2.3 Exclusion criteria

1. Studies with incomplete data or unreported data; (2) Non-RCTs (including quasi-RCTs, animal studies, protocols, conference abstracts, case reports or correspondence); (3) Study on mixed preparations of various botanical drugs or plant extracts; (4) Research on synthetic drugs with similar composition to botanical drugs or plant extracts.

### 2.4 Study selection

The EndNote reference management software was used to screen and exclude studies. Two researchers initially screened the titles of studies to identify duplicates, non-RCTs, review articles, conference papers, protocols, and correspondence. Subsequently, the abstracts of the studies were reviewed by the two researchers to determine eligible and ineligible studies. Finally, the full texts of the remaining studies were independently reviewed by the two researchers to further confirm the studies for inclusion. During this process, the two researchers independently conducted the screening and compared the remaining studies. If their decisions were consistent, the studies were included. In cases of discrepancies, a third researcher was consulted to resolve the issue through discussion.

### 2.5 Data extraction

This study utilized a standardized, pre-specified data extraction form with seven items to record the following information: (1) author, (2) publication year, (3) country, (4) study duration, (5) sample size, (6) mean age, and (7) details of the intervention.

### 2.6 Risk of bias in individual studies

Two researchers independently assessed the risk of bias (ROB) using the Cochrane Handbook version 5.1.0 to evaluate the ROB in RCTs. The assessment considered the following seven aspects: (1) generation of the random sequence; (2) allocation concealment; (3) blinding of participants; (4) blinding of personnel; (5) blinding of outcome assessors; (6) incomplete outcome data; and (7) selective reporting. Based on the number of metabolites with potentially high ROB, trials were categorized into three levels of ROB: high risk (five or more metabolites), moderate risk (three or four metabolites), and low risk (two or fewer metabolites) (Higgins et al., 2011).

### 2.7 Data analysis

In studies using botanical and medicinal plant extracts as interventions, all variables were continuous and were expressed as means with standard deviations (SD) (Li and Chen, 2021). Continuous variables were reported as either mean differences (MD, defined as the absolute difference between the means of the treatment and control groups, calculated using the same scale) or standardized mean differences (SMD, defined as the mean difference between groups divided by the standard deviation of outcomes among participants, used for pooling trial data from different scales) along with their 95% confidence intervals (CI) for analysis. The effect sizes are categorized as negligible (SMD <0.2), small (0.2–0.49), moderate (0.5–0.79), or significant (≥0.8). For interpretation, we apply the same evaluation criteria to the absolute values of SMD: negative SMD values (indicating better outcomes in the intervention group) are still assessed using the same evaluation standards after converting to absolute values. Given the potential heterogeneity across studies, a random-effects model was selected for analysis instead of a fixed-effects model (Jackson et al., 2011).

We used Stata software (version 15.1) in accordance with the PRISMA-NMA guidelines (Moher et al., 2015) to perform NMA aggregation and analysis within a Bayesian framework using Markov chain Monte Carlo (MCMC) simulation. To ensure the validity of the NMA, we carefully assessed transitivity and consistency, which are critical assumptions underlying indirect treatment comparisons. Transitivity was evaluated by examining the distribution of key effect modifiers (such as patient demographics, baseline severity of KOA, intervention doses, and study duration) across the included studies. We considered the assumption plausible if the populations, interventions, and outcomes were sufficiently similar to allow for meaningful indirect comparisons. Consistency was evaluated by comparing direct and indirect evidence using both local (node-splitting) and global inconsistency models. Statistical evidence for inconsistency was considered significant if p < 0.05. Deviations from transitivity or consistency assumptions were documented, and sensitivity analyses were performed to test the robustness of the results (Salanti et al., 2011).

Stata software was used to generate and describe network diagrams for different exercise interventions. Each node in the generated network diagram represents a specific exercise intervention or control condition, and the lines connecting the nodes represent direct head-to-head comparisons between interventions. The size of each node and the thickness of the connecting lines were proportional to the number of studies included (Chaimani et al., 2013).

The intervention hierarchy was summarized and reported using P-scores. P-scores are considered frequency simulations under the surface of the cumulative ranking curve (SUCRA) values, measuring the certainty of one treatment being better than another across all competing treatments, on average. The P-score ranges from 0 to 1, where a score of 1 indicates the best treatment with no uncertainty, and a score of 0 indicates the worst treatment with no uncertainty. Although P-scores or SUCRA values can effectively be reinterpreted as percentages representing the effectiveness or acceptability of interventions, these scores should be interpreted cautiously unless there are clinically meaningful differences between interventions. To examine potential bias in small-scale studies, which could lead to publication bias in the NMA, a network funnel plot was generated and visually inspected for symmetry (Khera et al., 2016).

### 2.8 GRADE evidence assessment

We applied the GRading of Recommendations Assessment, Development and Evaluation (GRADE) framework to rate the certainty of evidence for the three primary outcomes: knee pain intensity, knee stiffness severity, and limitation of knee function. RCTs begin as high-certainty evidence. We then evaluated five potential downgrade domains: (1) risk of bias, (2) inconsistency, (3) indirectness, (4) imprecision, and (5) publication bias, lowering the grade one level for each serious limitation identified. All included studies were RCTs with no serious risk of bias, indirectness, or imprecision; however, serious inconsistency was present for every outcome. Consequently, the certainty of evidence was downgraded by one level for each outcome, resulting in a final rating of moderate certainty.

## 3 Results

### 3.1 Study identification and selection

**FIGURE 1:**
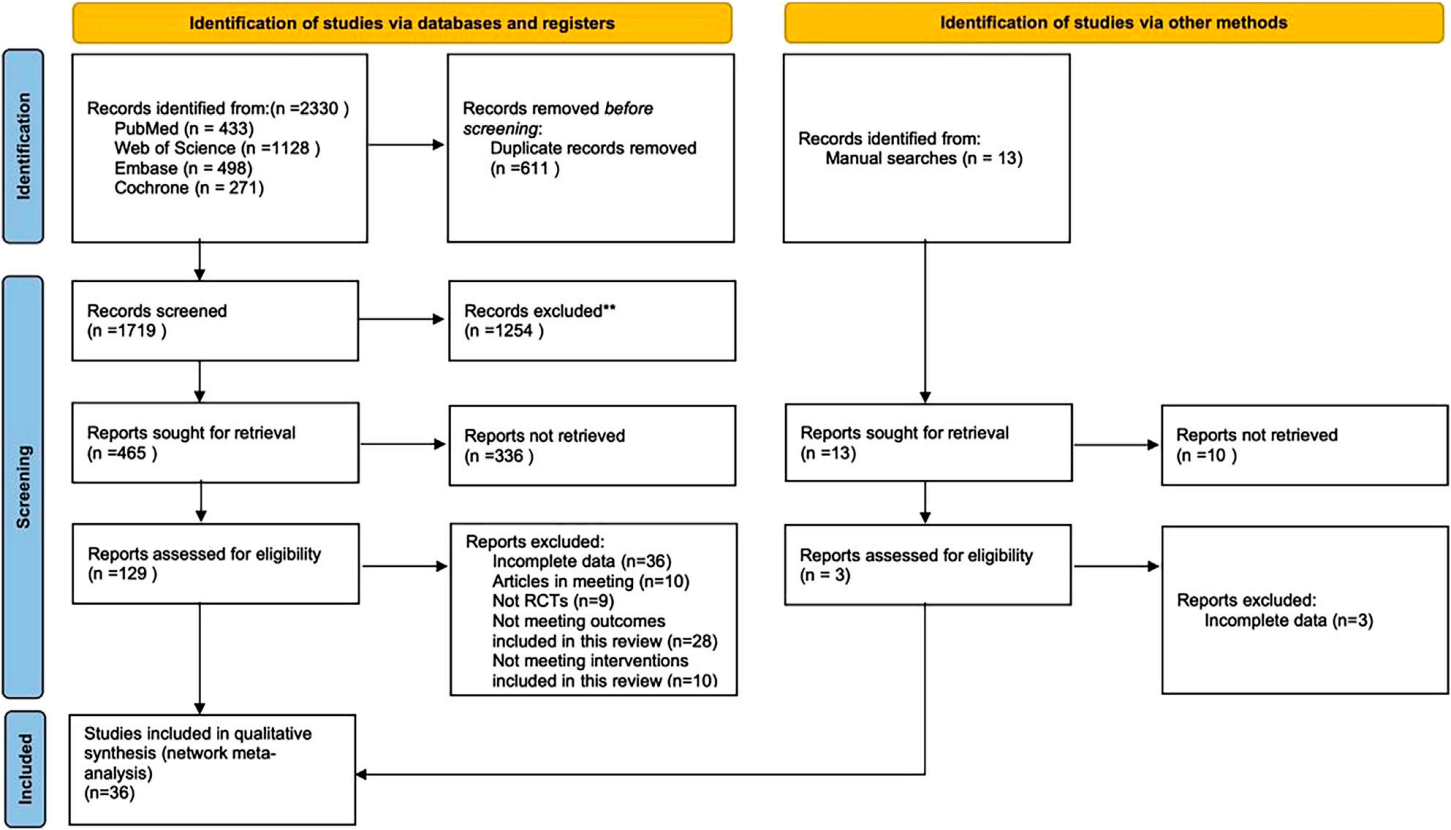
Flowchart of literature selection.

A total of 2,330 articles were retrieved from electronic databases, along with an additional 13 articles identified through manual searches. After removing duplicates, 1,732 articles remained for title and abstract screening, after which 1,603 were excluded. The remaining 129 articles underwent full-text review, resulting in the exclusion of another 93 articles for reasons including non-RCTs, incomplete data, conference papers, or failure to meet the inclusion criteria for interventions. Ultimately, 36 articles were included in this study (Figure 1).

### 3.2 Quality assessment of included studies

A total of 36 RCTs were included in this study. Overall, the quality of the included studies was moderate. Specific details will be presented in Table 1.

**TABLE 1:** Characteristics of the studies included in the meta-analysis.

Author	Country	Year	Age (mean + SD)	Total/male/female	Intervention	Control	Outcome
Altman	United States	2001	T: 64.0 (11.5)	T: 124/50/74	Ginger extract	Placebo	WOMAC, SF-36
			C: 66.3 (11.6)	C: 123/45/78	Length of Intervention: 6 weeks		
					Freq: 2 times a day		
					Single dose: 255 mg		
Wigler	Israel	2003	T: 64.7 (NA)	T: 14/1/13	Ginger extract	Placebo	VAS
			C: 59.3 (NA)	C: 15/5/10	Length of Intervention: 12 weeks		
					Freq: 4 times a day		
					Single dose: 250 mg		
Brien	United Kingdom	2006	T: 62.83 (9.36)	T: 24/13/11	Pineapple extract	Placebo	WOMAC,SF-36
			C: 60.43 (7.63)	C: 23/11/12	Length of Intervention: 12 weeks		
					Freq: 1 time a day		
					Single dose: 800 mg		
Sontakke	India	2007	T: NA	T: 33/NA/NA	Boswellia serrata extract	Usual treatment	WOMAC
			C: NA	C: 33/NA/NA	Length of Intervention: 6 months		
					Freq: NA		
					Single dose: NA		
Farid	Iran	2010	T: 55 (14.1)	T: 17/5/12	Passion fruit peel extract	Placebo	WOMAC
			C: 49.71 (14)	C: 16/3/13	Length of Intervention: 2 months		
					Freq: 1 time a day		
					Single dose: 150 mg		
Kuptniratsaikul	Thailand	2011	T: 59.4 (7)	T: 55/8/47	Derris scandens Benth extract	Usual treatment	WOMAC
			C: 60.5 (8.2)	C: 52/10/42	Length of Intervention: 4 weeks		
					Freq: 1 time a day		
					Single dose: 800 mg		
Zakeri	Iran	2011	T: 48.4 (11.1)	T: 103/20/83	Ginger extract	Placebo	WOMAC, VAS
			C: 45.74 (12.5)	C: 101/20/81	Length of Intervention: 6 weeks		
					Freq: 2 times a day		
					Single dose: 250 mg		
Pinsornsak	Thailand	2012	T: NA	T: 36/NA/NA	Curcuma extract	Usual treatment	VAS, KOOS
			C: NA	C: 37/NA/NA	Length of Intervention: 3 months		
					Freq: 2 times a day		
					Single dose: 500 mg		
Sadat	Iran	2013	T: NA	T: 22/NA/NA	Sesame extract	Usual treatment	VAS, KOOS
			C: NA	C: 23/NA/NA	Length of Intervention: 2 months		
					Freq: 1 time a day		
					Single dose: 40 g		
Madhu	India	2013	T: 56.63 (10.58)	T: 30/13/17	Curcuma extract	Placebo	WOMAC, VAS
			C: 56.77 (9.98)	C: 30/13/17	Length of Intervention: 42 days		
					Freq: 2 times a day		
					Single dose: 500 mg		
Schumacher	United States	2013	T: 56.7 (11.3)	T: 27/NA/NA	Cherry extract	Placebo	WOMAC
			C: 56.7 (11.3)	C: 31/NA/NA	Length of Intervention: 6 weeks		
					Freq: 1 time a day		
					Single dose: 8 oz		
Takeda	Japan	2013	T: 61.4 (8.3)	T: 13/3/10	Olive leave extract	Placebo	JOA
			C: 60.8 (7.2)	C: 12/3/9	Length of Intervention: 4 weeks		
					Freq: 1 time a day		
					Single dose: 50.1 mg		
Kuptniratsaikul	Thailand	2014	T: 60.3 (6.8)	T: 171/14/157	Curcuma extract	Usual treatment	WOMAC
			C: 60.9 (6.9)	C: 160/21/139	Length of Intervention: 4 weeks		
					Freq: 1 time a day		
					Single dose: 1500 mg		
Panahi	Iran	2014	T: 57.32 (8.78)	T: 27/5/22	Curcuma extract	Placebo	WOMAC
			C: 57.57 (9.05)	C: 26/4/22	Length of Intervention: 6 weeks		
					Freq: 3 times a week		
					Single dose: 500 mg		
Ghoochani	Iran	2016	T: 56.74 (10.23)	T: 19/2/17	Pomegranate extract	Placebo	WOMAC
			C: 53.84 (11.95)	C: 19/2/17	Length of Intervention: 6 weeks		
					Freq: 1 time a day		
					Single dose: 200 mL		
Panahi	Iran	2016	T: 55.2 (7.8)	T: 32/6/26	Elaeagnus Angustifolia extract	Usual treatment	WOMAC, VAS, LPFI
			C: 53.7 (10)	C: 32/2/30	Length of Intervention: 7 weeks		
					Freq: 1 time a day		
					Single dose: 600 mg		
Ramakanth	India	2016	T: 58.92 (6.07)	T: 20/13/7	Ashwagandha extract	Placebo	WOMAC, VAS
			C: 58.95 (3.73)	C: 20/16/4	Length of Intervention: 12 weeks		
					Freq: 2 times a week		
					Single dose: 250 mg		
Srivastava	India	2016	T: 50.23 (8.08)	T: 78/25/53	Curcuma extract	Placebo	WOMAC, VAS
			C: 50.27 (8.63)	C: 82/32/50	Length of Intervention: 4 months		
					Freq: 1 time a day		
					Single dose: 500 mg		
Essouiri	Morocco	2017	T: 58.24 (8.8)	T: 55/4/51	Argan extract	Placebo	WOMAC, VAS
			C: 58.85 (5.6)	C: 52/3/49	Length of Intervention: 8 weeks		
					Freq: 1 time a day		
					Single dose: 80 mL		
Hashempur	Iran	2018	T: 56.65 (8.07)	T: 20/17/3	Green tea extract	Usual treatment	WOMAC, VAS
			C: 53.05 (11.06)	C: 20/15/5	Length of Intervention: 4 weeks		
					Freq: 3 times a day		
					Single dose: 500 mg		
Kakuo	Japan	2018	T: 53.2 (6.3)	T: 27/12/15	Guava leaf extract	Placebo	JKOM
			C: 54.4 (7.5)	C: 26/9/17	Length of Intervention: 12 weeks		
					Freq: 1 time a day		
					Single dose: 1000 mg		
May	Malaysia	2018	T: 61.97 (9.19)	T: 38/NA/NA	Momordica charantia extract	Placebo	KOOS
			C: 57.81 (9.15)	C: 37/NA/NA	Length of Intervention: 3 months		
					Freq: 3 times a day		
					Single dose: 500 mg		
Nash	United Kingdom	2018	T: 51.9 (9)	T: 61/33/28	Cucumis sativus extract	Usual treatment	WOMAC, VAS
			C: 52.5 (5)	C: 61/29/32	Length of Intervention: 180 days		
					Freq: 2 times a day		
					Single dose: 10 mg		
Panda	India	2018	T: 55.2 (8.58)	T: 25/NA/NA	Curcuma extract	Placebo	WOMAC, VAS
			C: 53.12 (8.25)	C: 25/NA/NA	Length of Intervention: 12 weeks		
					Freq: 1 time a day		
					Single dose: 500 mg		
Salimzadeh	Iran	2018	T: NA	T: 39/NA/39	Garlic extract	Placebo	WOMAC
			C: NA	C: 37/NA/37	Length of Intervention: 12 weeks		
					Freq: 2 times a day		
					Single dose: 500 mg		
Hancke	India	2019	T: 55.3 (8.2)	T: 35/4/31	Andrographis paniculata extract	Placebo	WOMAC, SF-36
			C: 55.7 (7.7)	C: 36/9/27	Length of Intervention: 84 days		
					Freq: 2 times a day		
					Single dose: 300 mg		
Henrotin	Belgium	2019	T: 60.9 (9.78)	T: 49/10/39	Curcuma extract	Placebo	KOOS
			C: 63.3 (7.69)	C: 45/11/34	Length of Intervention: 3 months		
					Freq: 2 times a day		
					Single dose: 3 caps		
Alazadeh	Iran	2020	T: NA	T: 33/NA/33	Fennel extract	Placebo	WOMAC, VAS
			C: NA	C: 33/NA/33	Length of Intervention: 2 weeks		
					Freq: 2 times a day		
					Single dose: 400 mg		
Ha	Korea	2021	T: 60.57 (8.93)	T: 53/7/46	Chrysanthemum zawadskii extract	Placebo	WOMAC, VAS, SF-36
			C: 59.63 (8.05)	C: 57/6/51	Length of Intervention: 12 weeks		
					Freq: 1 time a day		
					Single dose: 600 mg		
Singhal	India	2021	T: 53.1 (10.9)	T: 73/20/53	Curcuma extract	Usual treatment	WOMAC
			C: 50.8 (9.9)	C: 71/17/54	Length of Intervention: 6 weeks		
					Freq: 2 times a day		
					Single dose: 500 mg		
Henrotin	Belgium	2022	T: 55.69 (1.25)	T: 64/21/43	Rubus idaeus leaf extract	Placebo	WOMAC, VAS, SF-36
			C: 52.93 (1.39)	C: 68/25/43	Length of Intervention: 12 weeks		
					Freq: 1 time a day		
					Single dose: 400 mg		
Huseini	Iran	2022	T: 59.6 (9.1)	T: 58/16/42	Nigella sativa extract	Placebo	WOMAC, VAS
			C: 63.3 (8.6)	C: 58/13/45	Length of Intervention: 1 month		
					Freq: 3 times a day		
					Single dose: 5 mL		
Kim	Korea	2023	T: 42.3 (12.6)	T: 40/14/26	Zisu extract	Placebo	WOMAC, VAS
			C: 34.5 (14.5)	C: 40/14/26	Length of Intervention: 8 weeks		
					Freq: 2 times a day		
					Single dose: 700 mg		
Nash	UNITED KINGDOM	2023	T: NA	T: 31/13/18	Cucumis sativus extract	Placebo	WOMAC, VAS, LPFI
			C: NA	C: 46/21/25	Length of Intervention: 180 days		
					Freq: 2 times a day		
					Single dose: 50 mg		
Navid	Iran	2023	T: 57.33 (8.85)	T: 52/23/29	Jadwar extract	Placebo	WOMAC, VAS
			C: 59.43 (7.88)	C: 52/24/28	Length of Intervention: 4 weeks		
					Freq: 2 times a day		
					Single dose: 500 mg		
Taheri	Iran	2023	T: 54.13 (7.53)	T: 43/10/33	Squill oxymel extract	Placebo	VAS, KOOS
			C: 5508.8 (7.12)	C: 45/8/37	Length of Intervention: 8 weeks		
					Freq: 1 time a day		
					Single dose: 10 mL		

CON: control group with Placebo or Usual treatment, T: experimental group, C: control group, WOMAC: Western Ontario and McMaster Universities Osteoarthritis Index, VAS: Visual Analogue Scale, SF-36: Short Form 36 Health Survey, KOOS: Knee injury and Osteoarthritis Outcome Score, LPFI: Lequesne抯 Pain-Function Index, JOA: Japanese Orthopaedic Association Score.

We used Grade to evaluate the evidence level of three indicators. Since there was no closed loop situation for all indicators, all inconsistent dimensions were downgraded. Specific details are presented. Specific details will be presented in Supplementary Table S1.

### 3.3 Characteristics of included studies

A total of 36 RCTs involving 3,285 patients diagnosed with KOA were included in this study. The interventions in the control group included *Zingiber officinale* Roscoe [Zingiberaceae; Zingiberis rhizoma] extract (3 studies) (Altman and Marcussen, 2001; Wigler et al., 2003; Zakeri et al., 2011), *Ananas comosus* (L.) Merr. [Bromeliaceae; Ananasis caulis] extract (1 study) (Brien et al., 2006), *Boswellia serrata* Roxb. ex Colebr. [Burseraceae; Olibanum indicum] extract (1 study) (Sontakke et al., 2007), *Passiflora edulis* Sims [Passifloraceae; Passiflorae edulis pericarpium] extract (1 study) (Farid et al., 2010), *Derris scandens* (Roxb.) Benth. [Fabaceae; Derridis scandentis caulis] extract (1 study) (Kuptniratsaikul et al., 2011), *Curcuma longa* L. [Zingiberaceae; Curcumae longae rhizoma] extract (8 studies) (Henrotin et al., 2019; Kuptniratsaikul et al., 2014; Madhu et al., 2013; Panahi et al., 2014; Panda et al., 2018; Pinsornsak and Niempoog, 2012; Singhal et al., 2021; Srivastava et al., 2016), *Sesamum indicum* L. [Pedaliaceae; Sesami semen] extract (1 study) (Eftekhar Sadat et al., 2013), *Prunus cerasus* L. [Rosaceae; Pruni cerasi fructus] extract (1 study) (Schumacher et al., 2013), *Olea europaea* L. [Oleaceae; Oleae folium] extract (1 study) (Takeda et al., 2013), *Punica granatum* L. [Lythraceae; Granati pericarpium] extract (1 study) (Ghoochani et al., 2016), *Elaeagnus angustifolia* L. [Elaeagnaceae; Elaeagni angustifoliae fructus] extract(1 study) (Panahi et al., 2016), *Withania somnifera* (L.) Dunal [Solanaceae; Withaniae radix] extract (1 study) (Ramakanth et al., 2016), *Argania spinosa* (L.) Skeels [Sapotaceae; Arganiae oleum] extract (1 study) (Essouiri et al., 2017), *Camellia sinensis* (L.) Kuntze [Theaceae; Theae folium] extract (1 study) (Hashempur et al., 2018), *Psidium guajava* L. [Myrtaceae; Psidii guajavae folium] extract (1 study) (Kakuo et al., 2018), *Momordica charantia* L. [Cucurbitaceae; Momordicae fructus] extract (1 study) (May et al., 2018), *Cucumis sativus* L. [Cucurbitaceae; Cucumeris sativi fructus] extract (2 studies) (Nash et al., 2018; Nash et al., 2023), *Allium sativum* L. [Amaryllidaceae; Allii sativi bulbus] extract (1 study) (Salimzadeh et al., 2018), *Andrographis paniculata* (Burm.f.) Nees [Acanthaceae; Andrographidis herba] extract (1 study) (Hancke et al., 2019), *Foeniculum vulgare* Mill. [Apiaceae; Foeniculi fructus] extract (1 study) (Alazadeh et al., 2020), *Chrysanthemum zawadskii* var. *latilobum* [Asteraceae; Chrysanthemi flos] extract (1 study) (Ha et al., 2021), *Rubus idaeus* L. [Rosaceae; Rubi idaei folium] extract (1 study) (Henrotin et al., 2022), *Nigella sativa* L. [Ranunculaceae; Nigellae semen] extract (1 study) (Huseini et al., 2022), *erilla frutescens* (L.) Britton [Lamiaceae; Perillae folium] extract (1 study) (Kim et al., 2023), *Delphinium denudatum* Wall. ex Hook. f. and Thomson [Ranunculaceae; Delphinii radix] extractt (1 study) (Navid et al., 2023), and *Drimia maritima* (L.) Stearn [Asparagaceae; Scillae bulbus] extract (1 study) (Taheri et al., 2023). Twenty-eight studies reported WOMAC as the outcome measure, 21 studies reported VAS, 5 studies reported KOOS, and 5 studies reported SF-36 as outcome measures. Of these, 28 studies were conducted in Asia, 2 in the Americas, 5 in Europe, and 1 in Africa. Since the age and gender distribution in the included studies are relatively consistent, transitivity is considered to be good. The characteristics of the included studies are summarized in Table 2. The pharmacopoeia and detailed characteristics of each plant extract are shown in Table 3.

**TABLE 2:** League table on Pain Score.

CS extract	Ashwagandha extract	AP extract	Jadwar extract	PFP extract	Curcuma extract	Sesame extract	MC extract	NS extract	Fennel extract	Ginger extract	Pomegranate extract	GL extract	Pineapple extract
CS extract	2.87 (−1.76,7.50)	4.85 (0.31,9.39)	4.99 (0.46,9.52)	5.01 (0.43,9.59)	5.55 (2.66,8.45)	5.50 (0.94,10.07)	5.59 (1.06,10.13)	5.79 (1.27,10.31)	5.87 (1.33,10.40)	5.99 (2.50,9.49)	6.13 (1.57,10.68)	6.18 (1.64,10.72)	6.36 (1.80,10.92)
−2.87 (−7.50,1.76)	Ashwagandha extract	1.98 (−3.13,7.09)	2.12 (−2.98,7.22)	2.14 (−3.00,7.28)	2.68 (−1.30,6.66)	2.63 (−2.98,8.25)	2.72 (−2.38,7.82)	2.92 (−2.17,8.01)	3.00 (−2.11,8.10)	3.12 (−1.08,7.33)	3.26 (−1.86,8.37)	3.31 (−1.80,8.42)	3.49 (−1.64,8.61)
−4.85 (−9.39,−0.31)	−1.98 (−7.09,3.13)	AP extract	0.14 (−4.87,5.16)	0.16 (−4.89,5.22)	0.70 (−3.17,4.57)	0.66 (−4.88,6.20)	0.75 (−4.27,5.77)	0.94 (−4.07,5.95)	1.02 (−4.00,6.04)	1.15 (−2.96,5.25)	1.28 (−3.76,6.32)	1.33 (−3.69,6.36)	1.51 (−3.53,6.55)
−4.99 (−9.52,−0.46)	−2.12 (−7.22,2.98)	−0.14 (−5.16,4.87)	Jadwar extract	0.02 (−5.02,5.07)	0.56 (−3.29,4.42)	0.52 (−5.01,6.04)	0.61 (−4.40,5.61)	0.80 (−4.20,5.80)	0.88 (−4.13,5.88)	1.00 (−3.09,5.09)	1.14 (−3.89,6.16)	1.19 (−3.82,6.20)	1.37 (−3.66,6.40)
−5.01 (−9.59,−0.43)	−2.14 (−7.28,3.00)	−0.16 (−5.22,4.89)	−0.02 (−5.07,5.02)	PFP extract	0.54 (−3.37,4.45)	0.49 (−5.07,6.06)	0.58 (−4.47,5.63)	0.78 (−4.26,5.82)	0.86 (−4.20,5.91)	0.98 (−3.16,5.13)	1.12 (−3.95,6.18)	1.17 (−3.89,6.23)	1.35 (−3.73,6.42)
−5.55 (−8.45,−2.66)	−2.68 (−6.66,1.30)	−0.70 (−4.57,3.17)	−0.56 (−4.42,3.29)	−0.54 (−4.45,3.37)	Curcuma extract	−0.05 (−4.08,3.99)	0.04 (−3.81,3.90)	0.24 (−3.61,4.09)	0.32 (−3.54,4.18)	0.44 (−2.12,3.00)	0.58 (−3.31,4.46)	0.63 (−3.24,4.50)	0.81 (−3.08,4.70)
−5.50 (−10.07,−0.94)	−2.63 (−8.25,2.98)	−0.66 (−6.20,4.88)	−0.52 (−6.04,5.01)	−0.49 (−6.06,5.07)	0.05 (−3.99,4.08)	Sesame extract	0.09 (−5.44,5.62)	0.29 (−5.24,5.81)	0.36 (−5.17,5.89)	0.49 (−4.23,5.20)	0.62 (−4.92,6.17)	0.68 (−4.86,6.21)	0.85 (−4.70,6.41)
−5.59 (−10.13,−1.06)	−2.72 (−7.82,2.38)	−0.75 (−5.77,4.27)	−0.61 (−5.61,4.40)	−0.58 (−5.63,4.47)	−0.04 (−3.90,3.81)	−0.09 (−5.62,5.44)	MC extract	0.20 (−4.80,5.20)	0.27 (−4.74,5.28)	0.40 (−3.69,4.49)	0.53 (−4.50,5.56)	0.59 (−4.43,5.60)	0.76 (−4.27,5.80)
−5.79 (−10.31,−1.27)	−2.92 (−8.01,2.17)	−0.94 (−5.95,4.07)	−0.80 (−5.80,4.20)	−0.78 (−5.82,4.26)	−0.24 (−4.09,3.61)	−0.29 (−5.81,5.24)	−0.20 (−5.20,4.80)	NS extract	0.08 (−4.93,5.08)	0.20 (−3.88,4.29)	0.34 (−4.68,5.36)	0.39 (−4.62,5.40)	0.57 (−4.46,5.59)
−5.87 (−10.40,−1.33)	−3.00 (−8.10,2.11)	−1.02 (−6.04,4.00)	−0.88 (−5.88,4.13)	−0.86 (−5.91,4.20)	−0.32 (−4.18,3.54)	−0.36 (−5.89,5.17)	−0.27 (−5.28,4.74)	−0.08 (−5.08,4.93)	Fennel extract	0.13 (−3.97,4.22)	0.26 (−4.77,5.29)	0.31 (−4.70,5.33)	0.49 (−4.54,5.53)
−5.99 (−9.49,−2.50)	−3.12 (−7.33,1.08)	−1.15 (−5.25,2.96)	−1.00 (−5.09,3.09)	−0.98 (−5.13,3.16)	−0.44 (−3.00,2.12)	−0.49 (−5.20,4.23)	−0.40 (−4.49,3.69)	−0.20 (−4.29,3.88)	−0.13 (−4.22,3.97)	Ginger extract	0.13 (−3.98,4.25)	0.19 (−3.91,4.29)	0.37 (−3.76,4.49)
−6.13 (−10.68,−1.57)	−3.26 (−8.37,1.86)	−1.28 (−6.32,3.76)	−1.14 (−6.16,3.89)	−1.12 (−6.18,3.95)	−0.58 (−4.46,3.31)	−0.62 (−6.17,4.92)	−0.53 (−5.56,4.50)	−0.34 (−5.36,4.68)	−0.26 (−5.29,4.77)	−0.13 (−4.25,3.98)	Pomegranate extract	0.05 (−4.98,5.09)	0.23 (−4.82,5.28)
−6.18 (−10.72,−1.64)	−3.31 (−8.42,1.80)	−1.33 (−6.36,3.69)	−1.19 (−6.20,3.82)	−1.17 (−6.23,3.89)	−0.63 (−4.50,3.24)	−0.68 (−6.21,4.86)	−0.59 (−5.60,4.43)	−0.39 (−5.40,4.62)	−0.31 (−5.33,4.70)	−0.19 (−4.29,3.91)	−0.05 (−5.09,4.98)	GL extract	0.18 (−4.86,5.22)
−6.36 (−10.92,−1.80)	−3.49 (−8.61,1.64)	−1.51 (−6.55,3.53)	−1.37 (−6.40,3.66)	−1.35 (−6.42,3.73)	−0.81 (−4.70,3.08)	−0.85 (−6.41,4.70)	−0.76 (−5.80,4.27)	−0.57 (−5.59,4.46)	−0.49 (−5.53,4.54)	−0.37 (−4.49,3.76)	−0.23 (−5.28,4.82)	−0.18 (−5.22,4.86)	Pineapple extract
−6.40 (−10.96,−1.85)	−3.53 (−9.14,2.07)	−1.56 (−7.08,3.97)	−1.42 (−6.93,4.10)	−1.39 (−6.95,4.16)	−0.85 (−4.88,3.17)	−0.90 (−5.92,4.12)	−0.81 (−6.33,4.71)	−0.61 (−6.12,4.90)	−0.54 (−6.06,4.98)	−0.41 (−5.11,4.29)	−0.28 (−5.81,5.26)	−0.22 (−5.75,5.30)	−0.05 (−5.59,5.49)
−6.34 (−10.87,−1.81)	−3.47 (−8.57,1.62)	−1.50 (−6.51,3.52)	−1.35 (−6.36,3.65)	−1.33 (−6.38,3.71)	−0.79 (−4.65,3.06)	−0.84 (−6.36,4.69)	−0.75 (−5.76,4.26)	−0.55 (−5.55,4.45)	−0.48 (−5.48,4.53)	−0.35 (−4.44,3.74)	−0.22 (−5.24,4.81)	−0.16 (−5.17,4.85)	0.02 (−5.02,5.05)
−6.38 (−10.90,−1.85)	−3.51 (−8.60,1.59)	−1.53 (−6.54,3.48)	−1.39 (−6.38,3.61)	−1.37 (−6.41,3.68)	−0.83 (−4.67,3.02)	−0.87 (−6.39,4.65)	−0.78 (−5.78,4.22)	−0.59 (−5.58,4.41)	−0.51 (−5.51,4.49)	−0.38 (−4.47,3.70)	−0.25 (−5.27,4.77)	−0.20 (−5.20,4.81)	−0.02 (−5.04,5.01)
−6.46 (−11.03,−1.89)	−3.59 (−9.20,2.03)	−1.61 (−7.15,3.93)	−1.47 (−7.00,4.06)	−1.45 (−7.01,4.12)	−0.91 (−4.95,3.13)	−0.95 (−5.99,4.08)	−0.86 (−6.39,4.67)	−0.67 (−6.19,4.86)	−0.59 (−6.12,4.94)	−0.46 (−5.18,4.25)	−0.33 (−5.88,5.22)	−0.28 (−5.81,5.26)	−0.10 (−5.65,5.46)
−6.44 (−10.99,−1.90)	−3.57 (−9.17,2.02)	−1.60 (−7.11,3.92)	−1.45 (−6.96,4.05)	−1.43 (−6.98,4.11)	−0.89 (−4.90,3.12)	−0.94 (−5.95,4.07)	−0.85 (−6.36,4.66)	−0.65 (−6.16,4.85)	−0.58 (−6.09,4.93)	−0.45 (−5.14,4.24)	−0.32 (−5.84,5.21)	−0.26 (−5.78,5.25)	−0.08 (−5.62,5.45)
−6.52 (−11.06,−1.97)	−3.65 (−8.76,1.46)	−1.67 (−6.70,3.36)	−1.53 (−6.54,3.49)	−1.51 (−6.57,3.55)	−0.97 (−4.84,2.91)	−1.01 (−6.55,4.53)	−0.92 (−5.94,4.10)	−0.73 (−5.74,4.28)	−0.65 (−5.67,4.37)	−0.52 (−4.63,3.58)	−0.39 (−5.43,4.65)	−0.34 (−5.36,4.69)	−0.16 (−5.20,4.89)
−6.53 (−11.09,−1.98)	−3.66 (−9.26,1.94)	−1.69 (−7.21,3.84)	−1.54 (−7.06,3.97)	−1.52 (−7.08,4.03)	−0.98 (−5.01,3.04)	−1.03 (−6.05,3.99)	−0.94 (−6.46,4.58)	−0.74 (−6.25,4.77)	−0.67 (−6.19,4.85)	−0.54 (−5.24,4.16)	−0.41 (−5.94,5.13)	−0.35 (−5.88,5.17)	−0.17 (−5.72,5.37)
−6.55 (−11.07,−2.03)	−3.68 (−8.77,1.41)	−1.70 (−6.71,3.30)	−1.56 (−6.55,3.43)	−1.54 (−6.58,3.50)	−1.00 (−4.84,2.84)	−1.04 (−6.56,4.47)	−0.96 (−5.95,4.04)	−0.76 (−5.75,4.23)	−0.68 (−5.68,4.32)	−0.56 (−4.64,3.52)	−0.42 (−5.44,4.59)	−0.37 (−5.37,4.63)	−0.19 (−5.21,4.83)
−6.44 (−9.31,−3.58)	−3.57 (−7.91,0.77)	−1.60 (−5.84,2.65)	−1.45 (−5.68,2.77)	−1.43 (−5.71,2.85)	−0.89 (−2.80,1.01)	−0.94 (−4.50,2.62)	−0.85 (−5.08,3.38)	−0.65 (−4.88,3.57)	−0.58 (−4.81,3.66)	−0.45 (−3.54,2.64)	−0.32 (−4.57,3.94)	−0.26 (−4.50,3.98)	−0.08 (−4.35,4.18)
−6.54 (−11.06,−2.02)	−3.67 (−8.76,1.42)	−1.69 (−6.70,3.31)	−1.55 (−6.55,3.44)	−1.53 (−6.57,3.51)	−0.99 (−4.84,2.86)	−1.04 (−6.56,4.48)	−0.95 (−5.95,4.05)	−0.75 (−5.74,4.24)	−0.67 (−5.68,4.33)	−0.55 (−4.63,3.53)	−0.42 (−5.43,4.60)	−0.36 (−5.37,4.64)	−0.18 (−5.21,4.84)
−6.61 (−11.13,−2.08)	−3.74 (−8.83,1.36)	−1.76 (−6.77,3.25)	−1.62 (−6.62,3.38)	−1.60 (−6.64,3.45)	−1.06 (−4.91,2.79)	−1.10 (−6.63,4.42)	−1.01 (−6.02,3.99)	−0.82 (−5.81,4.18)	−0.74 (−5.75,4.26)	−0.62 (−4.70,3.47)	−0.48 (−5.50,4.54)	−0.43 (−5.44,4.58)	−0.25 (−5.28,4.78)
−6.68 (−11.21,−2.15)	−3.81 (−8.91,1.29)	−1.83 (−6.85,3.18)	−1.69 (−6.69,3.31)	−1.67 (−6.71,3.38)	−1.13 (−4.98,2.73)	−1.17 (−6.70,4.35)	−1.08 (−6.09,3.92)	−0.89 (−5.89,4.11)	−0.81 (−5.82,4.20)	−0.68 (−4.77,3.40)	−0.55 (−5.58,4.47)	−0.50 (−5.51,4.51)	−0.32 (−5.35,4.71)
−6.80 (−11.37,−2.22)	−3.93 (−9.07,1.21)	−1.95 (−7.01,3.11)	−1.81 (−6.85,3.23)	−1.79 (−6.87,3.30)	−1.25 (−5.15,2.66)	−1.29 (−6.86,4.27)	−1.20 (−6.25,3.84)	−1.01 (−6.05,4.03)	−0.93 (−5.98,4.12)	−0.80 (−4.94,3.34)	−0.67 (−5.74,4.39)	−0.62 (−5.67,4.44)	−0.44 (−5.51,4.63)
−6.65 (−9.48,−3.83)	−3.78 (−7.45,−0.11)	−1.81 (−5.36,1.75)	−1.66 (−5.20,1.87)	−1.64 (−5.24,1.96)	−1.10 (−2.63,0.43)	−1.15 (−5.39,3.10)	−1.06 (−4.60,2.48)	−0.86 (−4.39,2.67)	−0.79 (−4.33,2.76)	−0.66 (−2.71,1.39)	−0.53 (−4.10,3.04)	−0.47 (−4.02,3.08)	−0.29 (−3.87,3.28)

BS extract	Zisu extract	Argan extract	GT extract	DSB extracts	Cherry extract	EA extract	RIL extract	Usual treatment	CZ extract	SO extract	Garlic extract	OL extract	Placebo
6.40 (1.85,10.96)	6.34 (1.81,10.87)	6.38 (1.85,10.90)	6.46 (1.89,11.03)	6.44 (1.90,10.99)	6.52 (1.97,11.06)	6.53 (1.98,11.09)	6.55 (2.03,11.07)	6.44 (3.58,9.31)	6.54 (2.02,11.06)	6.61 (2.08,11.13)	6.68 (2.15,11.21)	6.80 (2.22,11.37)	6.65 (3.83,9.48)
3.53 (−2.07,9.14)	3.47 (−1.62,8.57)	3.51 (−1.59,8.60)	3.59 (−2.03,9.20)	3.57 (−2.02,9.17)	3.65 (−1.46,8.76)	3.66 (−1.94,9.26)	3.68 (−1.41,8.77)	3.57 (−0.77,7.91)	3.67 (−1.42,8.76)	3.74 (−1.36,8.83)	3.81 (−1.29,8.91)	3.93 (−1.21,9.07)	3.78 (0.11,7.45)
1.56 (−3.97,7.08)	1.50 (−3.52,6.51)	1.53 (−3.48,6.54)	1.61 (−3.93,7.15)	1.60 (−3.92,7.11)	1.67 (−3.36,6.70)	1.69 (−3.84,7.21)	1.70 (−3.30,6.71)	1.60 (−2.65,5.84)	1.69 (−3.31,6.70)	1.76 (−3.25,6.77)	1.83 (−3.18,6.85)	1.95 (−3.11,7.01)	1.81 (−1.75,5.36)
1.42 (−4.10,6.93)	1.35 (−3.65,6.36)	1.39 (−3.61,6.38)	1.47 (−4.06,7.00)	1.45 (−4.05,6.96)	1.53 (−3.49,6.54)	1.54 (−3.97,7.06)	1.56 (−3.43,6.55)	1.45 (−2.77,5.68)	1.55 (−3.44,6.55)	1.62 (−3.38,6.62)	1.69 (−3.31,6.69)	1.81 (−3.23,6.85)	1.66 (−1.87,5.20)
1.39 (−4.16,6.95)	1.33 (−3.71,6.38)	1.37 (−3.68,6.41)	1.45 (−4.12,7.01)	1.43 (−4.11,6.98)	1.51 (−3.55,6.57)	1.52 (−4.03,7.08)	1.54 (−3.50,6.58)	1.43 (−2.85,5.71)	1.53 (−3.51,6.57)	1.60 (−3.45,6.64)	1.67 (−3.38,6.71)	1.79 (−3.30,6.87)	1.64 (−1.96,5.24)
0.85 (−3.17,4.88)	0.79 (−3.06,4.65)	0.83 (−3.02,4.67)	0.91 (−3.13,4.95)	0.89 (−3.12,4.90)	0.97 (−2.91,4.84)	0.98 (−3.04,5.01)	1.00 (−2.84,4.84)	0.89 (−1.01,2.80)	0.99 (−2.86,4.84)	1.06 (−2.79,4.91)	1.13 (−2.73,4.98)	1.25 (−2.66,5.15)	1.10 (−0.43,2.63)
0.90 (−4.12,5.92)	0.84 (−4.69,6.36)	0.87 (−4.65,6.39)	0.95 (−4.08,5.99)	0.94 (−4.07,5.95)	1.01 (−4.53,6.55)	1.03 (−3.99,6.05)	1.04 (−4.47,6.56)	0.94 (−2.62,4.50)	1.04 (−4.48,6.56)	1.10 (−4.42,6.63)	1.17 (−4.35,6.70)	1.29 (−4.27,6.86)	1.15 (−3.10,5.39)
0.81 (−4.71,6.33)	0.75 (−4.26,5.76)	0.78 (−4.22,5.78)	0.86 (−4.67,6.39)	0.85 (−4.66,6.36)	0.92 (−4.10,5.94)	0.94 (−4.58,6.46)	0.96 (−4.04,5.95)	0.85 (−3.38,5.08)	0.95 (−4.05,5.95)	1.01 (−3.99,6.02)	1.08 (−3.92,6.09)	1.20 (−3.84,6.25)	1.06 (−2.48,4.60)
0.61 (−4.90,6.12)	0.55 (−4.45,5.55)	0.59 (−4.41,5.58)	0.67 (−4.86,6.19)	0.65 (−4.85,6.16)	0.73 (−4.28,5.74)	0.74 (−4.77,6.25)	0.76 (−4.23,5.75)	0.65 (−3.57,4.88)	0.75 (−4.24,5.74)	0.82 (−4.18,5.81)	0.89 (−4.11,5.89)	1.01 (−4.03,6.05)	0.86 (−2.67,4.39)
0.54 (−4.98,6.06)	0.48 (−4.53,5.48)	0.51 (−4.49,5.51)	0.59 (−4.94,6.12)	0.58 (−4.93,6.09)	0.65 (−4.37,5.67)	0.67 (−4.85,6.19)	0.68 (−4.32,5.68)	0.58 (−3.66,4.81)	0.67 (−4.33,5.68)	0.74 (−4.26,5.75)	0.81 (−4.20,5.82)	0.93 (−4.12,5.98)	0.79 (−2.76,4.33)
0.41 (−4.29,5.11)	0.35 (−3.74,4.44)	0.38 (−3.70,4.47)	0.46 (−4.25,5.18)	0.45 (−4.24,5.14)	0.52 (−3.58,4.63)	0.54 (−4.16,5.24)	0.56 (−3.52,4.64)	0.45 (−2.64,3.54)	0.55 (−3.53,4.63)	0.62 (−3.47,4.70)	0.68 (−3.40,4.77)	0.80 (−3.34,4.94)	0.66 (−1.39,2.71)
0.28 (−5.26,5.81)	0.22 (−4.81,5.24)	0.25 (−4.77,5.27)	0.33 (−5.22,5.88)	0.32 (−5.21,5.84)	0.39 (−4.65,5.43)	0.41 (−5.13,5.94)	0.42 (−4.59,5.44)	0.32 (−3.94,4.57)	0.42 (−4.60,5.43)	0.48 (−4.54,5.50)	0.55 (−4.47,5.58)	0.67 (−4.39,5.74)	0.53 (−3.04,4.10)
0.22 (−5.30,5.75)	0.16 (−4.85,5.17)	0.20 (−4.81,5.20)	0.28 (−5.26,5.81)	0.26 (−5.25,5.78)	0.34 (−4.69,5.36)	0.35 (−5.17,5.88)	0.37 (−4.63,5.37)	0.26 (−3.98,4.50)	0.36 (−4.64,5.37)	0.43 (−4.58,5.44)	0.50 (−4.51,5.51)	0.62 (−4.44,5.67)	0.47 (−3.08,4.02)
0.05 (−5.49,5.59)	−0.02 (−5.05,5.02)	0.02 (−5.01,5.04)	0.10 (−5.46,5.65)	0.08 (−5.45,5.62)	0.16 (−4.89,5.20)	0.17 (−5.37,5.72)	0.19 (−4.83,5.21)	0.08 (−4.18,4.35)	0.18 (−4.84,5.21)	0.25 (−4.78,5.28)	0.32 (−4.71,5.35)	0.44 (−4.63,5.51)	0.29 (−3.28,3.87)
BS extract	−0.06 (−5.58,5.45)	−0.03 (−5.54,5.48)	0.05 (−4.97,5.08)	0.04 (−4.96,5.04)	0.11 (−5.41,5.64)	0.13 (−4.88,5.14)	0.14 (−5.36,5.65)	0.04 (−3.50,3.58)	0.14 (−5.37,5.65)	0.20 (−5.31,5.72)	0.27 (−5.24,5.79)	0.39 (−5.16,5.95)	0.25 (−3.98,4.48)
0.06 (−5.45,5.58)	Zisu extract	0.03 (−4.97,5.03)	0.11 (−5.41,5.64)	0.10 (−5.41,5.61)	0.17 (−4.84,5.19)	0.19 (−5.33,5.71)	0.21 (−4.79,5.20)	0.10 (−4.13,4.33)	0.20 (−4.80,5.19)	0.27 (−4.73,5.27)	0.33 (−4.67,5.34)	0.45 (−4.59,5.50)	0.31 (−3.23,3.85)
0.03 (−5.48,5.54)	−0.03 (−5.03,4.97)	Argan extract	0.08 (−5.44,5.61)	0.07 (−5.44,5.57)	0.14 (−4.87,5.15)	0.16 (−5.35,5.67)	0.17 (−4.82,5.16)	0.07 (−4.15,4.29)	0.17 (−4.83,5.16)	0.23 (−4.76,5.23)	0.30 (−4.70,5.30)	0.42 (−4.62,5.46)	0.28 (−3.25,3.81)
−0.05 (−5.08,4.97)	−0.11 (−5.64,5.41)	−0.08 (−5.61,5.44)	GT extract	−0.01 (−5.03,5.00)	0.06 (−5.48,5.60)	0.08 (−4.95,5.10)	0.09 (−5.43,5.61)	−0.01 (−3.58,3.55)	0.08 (−5.44,5.61)	0.15 (−5.37,5.68)	0.22 (−5.31,5.75)	0.34 (−5.23,5.91)	0.20 (−4.05,4.44)
−0.04 (−5.04,4.96)	−0.10 (−5.61,5.41)	−0.07 (−5.57,5.44)	0.01 (−5.00,5.03)	DSB extracts	0.07 (−5.45,5.59)	0.09 (−4.91,5.09)	0.11 (−5.39,5.60)	0.00 (−3.53,3.53)	0.10 (−5.40,5.60)	0.17 (−5.34,5.67)	0.23 (−5.27,5.74)	0.35 (−5.19,5.90)	0.21 (−4.01,4.43)
−0.11 (−5.64,5.41)	−0.17 (−5.19,4.84)	−0.14 (−5.15,4.87)	−0.06 (−5.60,5.48)	−0.07 (−5.59,5.45)	Cherry extract	0.02 (−5.51,5.54)	0.03 (−4.98,5.04)	−0.07 (−4.32,4.17)	0.02 (−4.99,5.03)	0.09 (−4.92,5.10)	0.16 (−4.86,5.18)	0.28 (−4.78,5.34)	0.14 (−3.42,3.69)
−0.13 (−5.14,4.88)	−0.19 (−5.71,5.33)	−0.16 (−5.67,5.35)	−0.08 (−5.10,4.95)	−0.09 (−5.09,4.91)	−0.02 (−5.54,5.51)	EA extract	0.02 (−5.49,5.52)	−0.09 (−3.63,3.45)	0.01 (−5.50,5.52)	0.08 (−5.44,5.59)	0.14 (−5.37,5.66)	0.26 (−5.29,5.82)	0.12 (−4.11,4.35)
−0.14 (−5.65,5.36)	−0.21 (−5.20,4.79)	−0.17 (−5.16,4.82)	−0.09 (−5.61,5.43)	−0.11 (−5.60,5.39)	−0.03 (−5.04,4.98)	−0.02 (−5.52,5.49)	RIL extract	−0.11 (−4.32,4.11)	−0.01 (−5.00,4.98)	0.06 (−4.93,5.05)	0.13 (−4.87,5.12)	0.25 (−4.79,5.28)	0.10 (−3.42,3.63)
−0.04 (−3.58,3.50)	−0.10 (−4.33,4.13)	−0.07 (−4.29,4.15)	0.01 (−3.55,3.58)	−0.00 (−3.53,3.53)	0.07 (−4.17,4.32)	0.09 (−3.45,3.63)	0.11 (−4.11,4.32)	Usual treatment	0.10 (−4.12,4.32)	0.17 (−4.06,4.39)	0.23 (−3.99,4.46)	0.35 (−3.92,4.63)	0.21 (−2.10,2.53)
−0.14 (−5.65,5.37)	−0.20 (−5.19,4.80)	−0.17 (−5.16,4.83)	−0.08 (−5.61,5.44)	−0.10 (−5.60,5.40)	−0.02 (−5.03,4.99)	−0.01 (−5.52,5.50)	0.01 (−4.98,5.00)	−0.10 (−4.32,4.12)	CZ extract	0.07 (−4.93,5.06)	0.14 (−4.86,5.13)	0.26 (−4.78,5.29)	0.11 (−3.42,3.64)
−0.20 (−5.72,5.31)	−0.27 (−5.27,4.73)	−0.23 (−5.23,4.76)	−0.15 (−5.68,5.37)	−0.17 (−5.67,5.34)	−0.09 (−5.10,4.92)	−0.08 (−5.59,5.44)	−0.06 (−5.05,4.93)	−0.17 (−4.39,4.06)	−0.07 (−5.06,4.93)	SO extract	0.07 (−4.93,5.07)	0.19 (−4.85,5.23)	0.04 (−3.49,3.58)
−0.27 (−5.79,5.24)	−0.33 (−5.34,4.67)	−0.30 (−5.30,4.70)	−0.22 (−5.75,5.31)	−0.23 (−5.74,5.27)	−0.16 (−5.18,4.86)	−0.14 (−5.66,5.37)	−0.13 (−5.12,4.87)	−0.23 (−4.46,3.99)	−0.14 (−5.13,4.86)	−0.07 (−5.07,4.93)	Garlic extract	0.12 (−4.92,5.16)	−0.02 (−3.56,3.51)
−0.39 (−5.95,5.16)	−0.45 (−5.50,4.59)	−0.42 (−5.46,4.62)	−0.34 (−5.91,5.23)	−0.35 (−5.90,5.19)	−0.28 (−5.34,4.78)	−0.26 (−5.82,5.29)	−0.25 (−5.28,4.79)	−0.35 (−4.63,3.92)	−0.26 (−5.29,4.78)	−0.19 (−5.23,4.85)	−0.12 (−5.16,4.92)	OL extract	−0.14 (−3.74,3.45)
−0.25 (−4.48,3.98)	−0.31 (−3.85,3.23)	−0.28 (−3.81,3.25)	−0.20 (−4.44,4.05)	−0.21 (−4.43,4.01)	−0.14 (−3.69,3.42)	−0.12 (−4.35,4.11)	−0.10 (−3.63,3.42)	−0.21 (−2.53,2.10)	−0.11 (−3.64,3.42)	−0.04 (−3.58,3.49)	0.02 (−3.51,3.56)	0.14 (−3.45,3.74)	Placebo

**TABLE 3:** Characteristics of extracts included in the literature.

Plant species	Pharmacopoeial status	Extract details (As reported in study)	Metabolites analyzed/quantified in original study	Reference
Foeniculum vulgare Mill	JP, EP (fruit/seed)	200 mg dried extract per capsule (from 7 g seeds), 70% ethanol maceration, 4 capsules/day	Total flavonoids 0.81 mg/g (quercetin equivalent)	Alazadeh et al., 2020
Zingiber officinale Roscoe (Ginger)	JP, USP, EP, BP	255 mg EV.EXT77 capsule twice daily (total 510 mg/day), extracted from 2.5–4 g dried ginger + 0.5–1.5 g Alpinia galanga, standardized to gingerols	Gingerols identified; no quantification of individual gingerols or total gingerol content reported	Altman and Marcussen, 2001
Delphinium denudatum Wall. (Jadwar)	No pharmacopoeial monograph identified	Root powder extract	Delphinine, stahisagrine, delphocurarine, condelphine, denudatin, isotalatizidine, quercetin	Navid et al., 2023
Ananas comosus (Bromelain)	USP	800 mg/day bromelain (two 200 mg tablets bid), crude aqueous pineapple stem extract, standardized enzyme activity (FIP units).	No quantification of individual enzymes or proteolytic activity reported.	Brien et al., 2006
Sesamum indicum L. (Sesame)	USP, EP (seeds/oil)	Ground sesame seed powder, 40 g/day orally for 2 months	Lignans (e.g., sesamin, sesamolin), vitamin E, unsaturated fatty acids (not quantified)	Eftekhar Sadat et al., 2013
Argania spinosa (Argan)	EP (oil)	Culinary argan oil, 30 mL/day for 8 weeks	Tocopherols (e.g., γ-tocopherol 75%), phytosterols, squalene, unsaturated fatty acids (reported from prior compositional analysis)	Essouiri et al., 2017
Passiflora edulis Sims (Passion fruit peel)	No pharmacopoeial monograph identified	150 mg/day PFP extract capsule, flavonoid-rich (anthocyanins, quercetin, luteolin, cyanidin-3-O-glucoside)	Flavonoids identified by HPLC; no quantification of individual flavonoids or total flavonoid content.	Farid et al., 2010
Punica granatum L. (Pomegranate)	EP (peel); juice not monographed	Pomegranate juice, 200 mL/day for 6 weeks	Polyphenols (punicalagin, ellagic acid), anthocyanins (not quantified in this study)	Ghoochani et al., 2016
Chrysanthemum zawadskii var. latilobum	Chinese Pharmacopoeia (Chrysanthemum morifolium); not in JP/USP/EP/BP	Ethanol extract from stems and leaves	Linarin, other flavonoids and phenols	Ha et al., 2021
Andrographis paniculata Nees	Chinese/Indian Pharmacopoeia; not in JP/USP/EP/BP	300 or 600 mg/day ParActin®, 50% andrographolide	Andrographolide quantified; 14-deoxyandrographolide & neoandrographolide also detected	Hancke et al., 2019
Camellia sinensis (Green tea)	JP, USP, EP, BP	1500 mg/day green tea tablet, 33.97% EGCG, 50.65 mg pyrogallol	EGCG and pyrogallol quantified by HPLC	Hashempur et al., 2018
Rubus idaeus (Raspberry)	No pharmacopoeial monograph identified	Ethanolic leaf extract	Sanguiine H6 (a polyphenol), other polyphenols	Henrotin et al., 2022
Curcuma longa L. (Turmeric)	JP, EP, USP (rhizome)	Bio-optimized Curcuma longa extract (Flexofytol), 46.67 mg turmeric rhizome extract per capsule with polysorbate 80 (E433) as emulsifier; low dose: 2脳2 capsules/day, high dose: 2脳3 capsules/day, duration 3 months	Curcumin serum levels quantified (pharmacokinetics assessed); sColl2-1 biomarker of cartilage degradation measured (ELISA); no detailed quantification of curcuminoid composition reported	Henrotin et al., 2019
Nigella sativa (Black Seed)	USP	Oil extracted from seeds	Thymoquinone, linoleic acid, palmitic acid, oleic acid	Huseini et al., 2022
Psidium guajava L. (Guava leaf)	No pharmacopoeial monograph identified	1 g/day guava leaf extract capsule	Ellagic acid identified; no total polyphenol content reported	Kakuo et al., 2018
Perilla frutescens (L.) Britton var. frutescens	JP (Perilla leaf), Chinese Pharmacopoeia	Extract from leaves	Isoegomaketone, perillaldehyde, anthocyanins, terpenoids, coumarins	Kim et al., 2023
Derris scandens Benth.	No pharmacopoeial monograph identified	800 mg/day of dried ethanolic extract (50% ethanol, reflux), standardized capsule (400 mg each), genistein-7-O-[rhamnopyranosyl-(1→6)-glucopyranoside] as major active.	Genistein derivative quantified by UPLC; no total isoflavonoid content reported.	Kuptniratsaikul et al., 2011
Curcuma domestica Valeton (Turmeric)	Same as Curcuma longa (JP, USP, EP, BP)	Curcuma domestica extract, 1500 mg/day	Curcuminoids 75–85% (curcumin:demethoxycurcumin:bisdemethoxycurcumin = 1:0.59:0.23)	Kuptniratsaikul et al., 2014
Curcuma longa L. (Turmeric)	JP, USP, EP, BP	500 mg NR-INF-02 capsule twice daily (total 1,000 mg/day), polysaccharide-rich extract (12.6% w/w polysaccharides by HPLC)	Polysaccharide content quantified; no curcuminoid analysis (curcuminoids explicitly removed)	Madhu et al., 2013
Cucumis sativus L. (Cucumber)	No pharmacopoeial monograph identified	10 mg twice daily Q-Actin™ aqueous extract, standardized to 1% IdoBR1	IdoBR1 iminosugar quantified	Nash et al., 2018
Cucumis sativus (Cucumber)	No pharmacopoeial monograph identified	Standardized extract with iminosugar idoBR1	Iminosugar idoBR1	Nash et al., 2023
Curcuma longa L. (Turmeric)	JP, USP, EP, BP	Curcuminoids extract (C3 Complex®), 1500 mg/day in 3 divided doses	Curcuminoids ≥95% (curcumin, demethoxycurcumin, bisdemethoxycurcumin); Bioperine® 5 mg/capsule	Panahi et al., 2014
Elaeagnus angustifolia L. (Russian Olive)	No pharmacopoeial monograph identified	Aqueous extract syrup, 300 or 600 mg/day for 7 weeks	Kaempferol 0.21% w/w (HPLC quantified); flavonoids and tannins (not fully quantified)	Panahi et al., 2016
Curcuma longa L.	JP, USP, EP, BP	500 mg/day Curene® capsule, Aquasome™ bioavailable curcuminoids	Curcuminoids (no total content specified)	Panda et al., 2018
Curcuma longa L. (Turmeric)	JP, USP, EP, BP	1,000 mg/day curcuminoid capsules (2×250 mg bid), combined with diclofenac 75 mg/day	Each capsule contained 250 mg turmeric extract equivalent to curcuminoids; no individual curcuminoid quantification	Pinsornsak and Niempoog, 2012
Withania somnifera (Ashwagandha)	USP	Aqueous extract of root + leaf (Sensoril®), 125 or 250 mg twice daily	Withanolide glycosides ≥10%, oligosaccharides ≥32%, Withaferin-A ≤0.5% (HPLC standardized)	Ramakanth et al., 2016
Allium sativum L. (Garlic)	BP, EP, USP	1000 mg/day odorless garlic tablet, 2.5 mg allicin	Allicin quantified; no total sulfur compound content reported	Salimzadeh et al., 2018
Prunus cerasus L. (Tart Cherry)	No pharmacopoeial monograph identified	Tart cherry juice blend (≥90% cherry juice), 8 oz twice daily for 6 weeks	Phenolics ≥450 mg/bottle (as gallic acid equivalents); Anthocyanins ≥30 mg/bottle (as cyanidin-3-glucoside)	Schumacher et al., 2013
Curcuma longa (Turmeric)	JP, USP, EP, BP	BCM-95 extract containing curcuminoids and essential oil of turmeric	Curcumin, demethoxycurcumin, bisdemethoxycurcumin, ar-turmerone	Singhal et al., 2021
Boswellia serrata Roxb. (Indian Frankincense)	EP (resin)	333 mg Boswellia serrata extract capsule thrice daily (total 999 mg/day), standardized to ≥40% total boswellic acids	Total boswellic acids quantified; individual acids reported (11-keto-β-BA: 6.44%, 3-O-acetyl-β-BA: 8.58%, etc.)	Sontakke et al., 2007
Momordica charantia L. (Bitter melon)	Chinese Pharmacopoeia; not in JP/USP/EP/BP	4500 mg/day whole fruit extract, 500 mg/capsule, 3 capsules TID	No specific compound quantified; phytochemical screening mentioned	May et al., 2018
Curcuma longa L. (Turmeric)	JP, USP, EP, BP	CL extract capsule (500 mg, 2× daily) for 4 months	≥95% curcuminoids (curcumin, demethoxycurcumin, bisdemethoxycurcumin)	Srivastava et al., 2016
Drimia maritima (Squill)	EP, USP	Oxymel syrup from squill vinegar extract and honey	Bufadienolides (e.g., proscillaridin A)	Taheri et al., 2023
Olea europaea L. (Olive leaf)	EP, USP (leaf/oil); hydroxytyrosol not monographed	50.1 mg/day olive extract (HT-20), containing 10.04 mg hydroxytyrosol	Hydroxytyrosol quantified by HPLC (22% of extract); no other phenolics quantified	Takeda et al., 2013
Zingiber officinale (Ginger)	JP, USP, EP, BP	250 mg Zintona EC capsule qid (total 1,000 mg/day), enteric-coated, supercritical CO₂ extract, 10 mg gingerol per capsule	Gingerol content quantified by HPLC; dissolution tested (20% release in gastric fluid, remainder in intestinal fluid)	Wigler et al., 2003
Zingiber officinale (Ginger)	JP, USP, EP, BP	250 mg Zintoma capsule twice daily (total 500 mg/day), containing 250 mg powdered ginger per capsule	No phytochemical quantification; only stated as “powdered ginger”	Zakeri et al., 2011

### 3.4 Network meta-analysis

**FIGURE 2:**
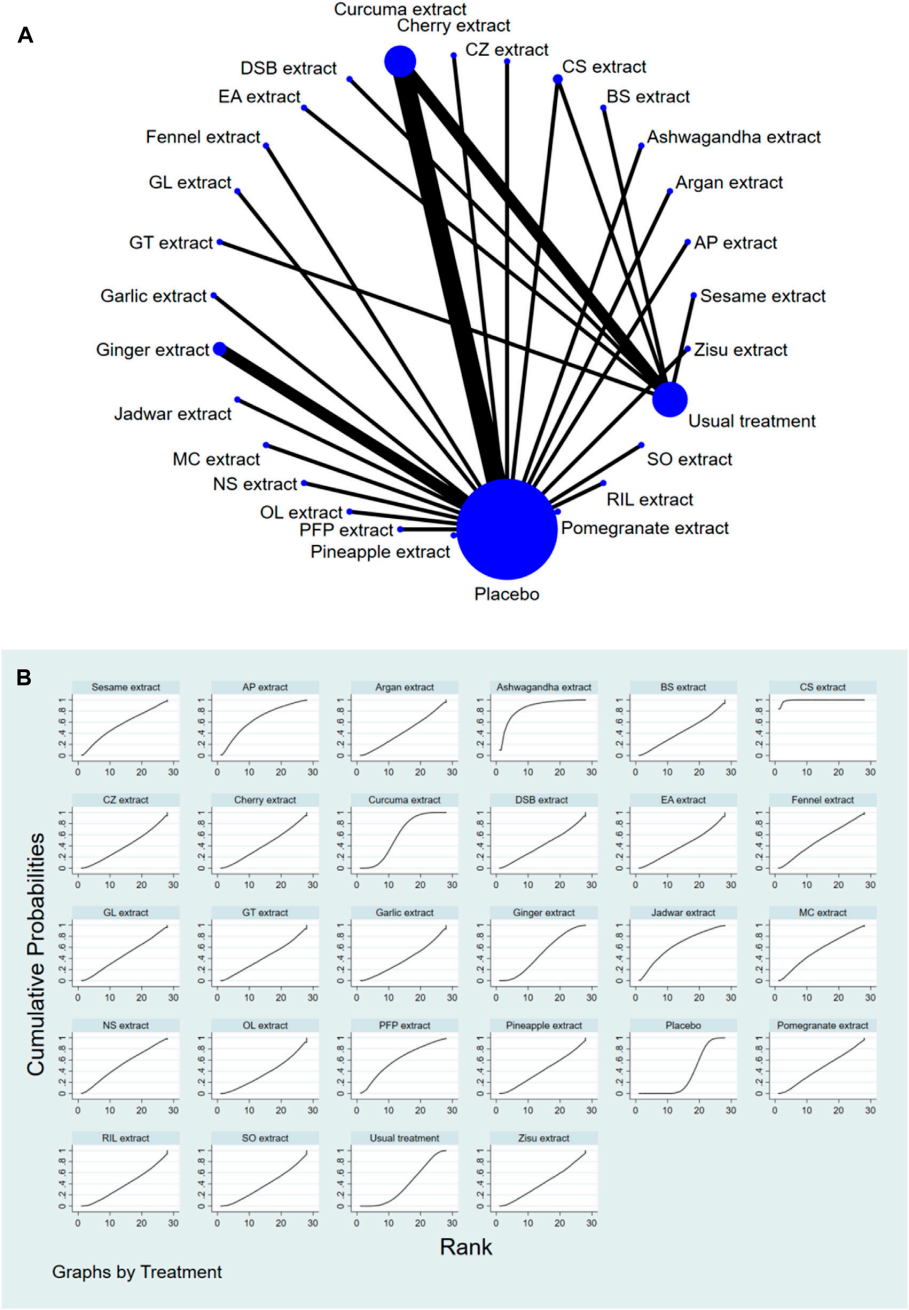
**(A)** NMA figure for Pain Score. **(B)** SUCRA plot for Pain Score.

**FIGURE 3:**
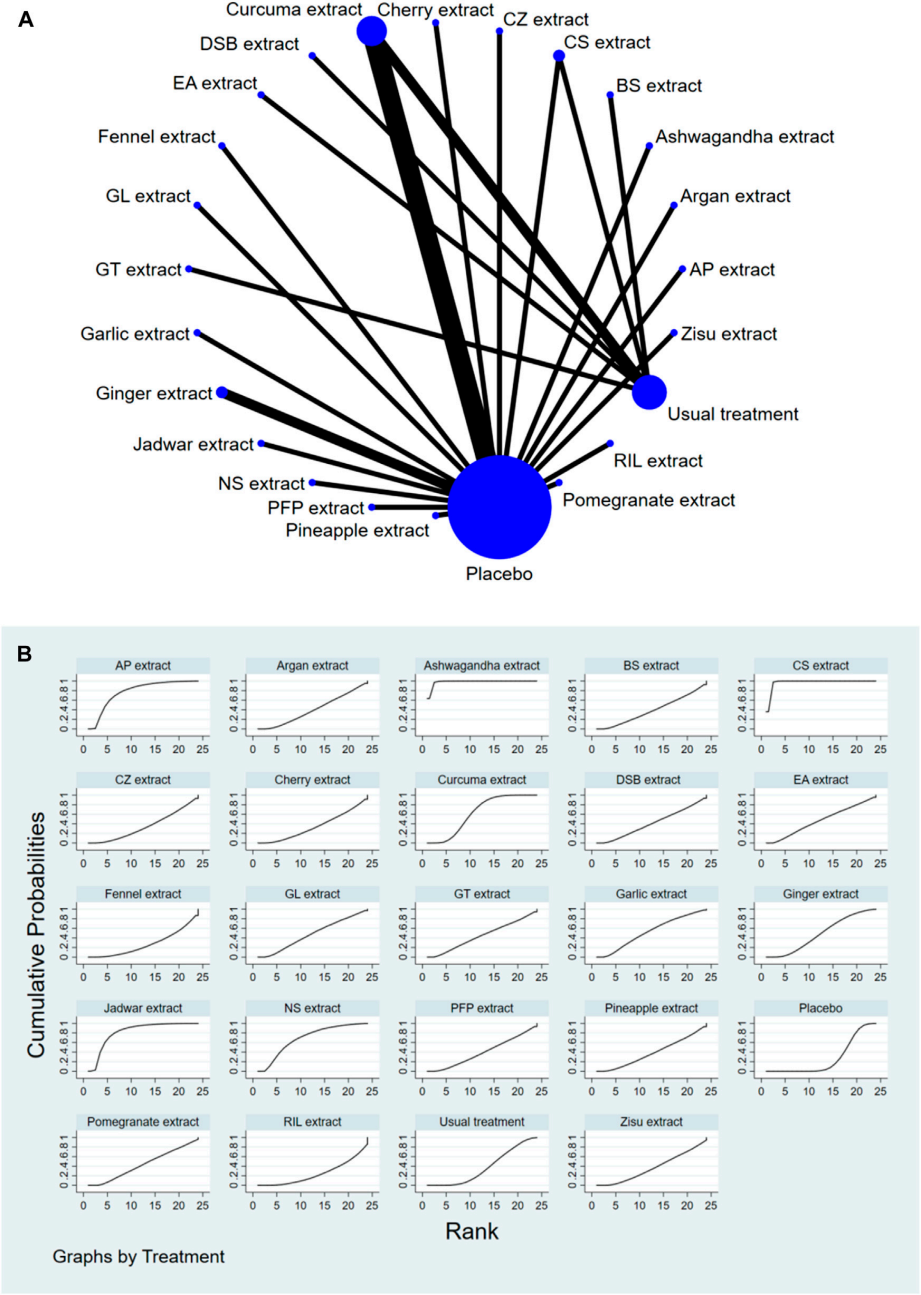
**(A)** NMA figure for Stiffness Score. **(B)** SUCRA plot for Stiffness Score.

**FIGURE 4:**
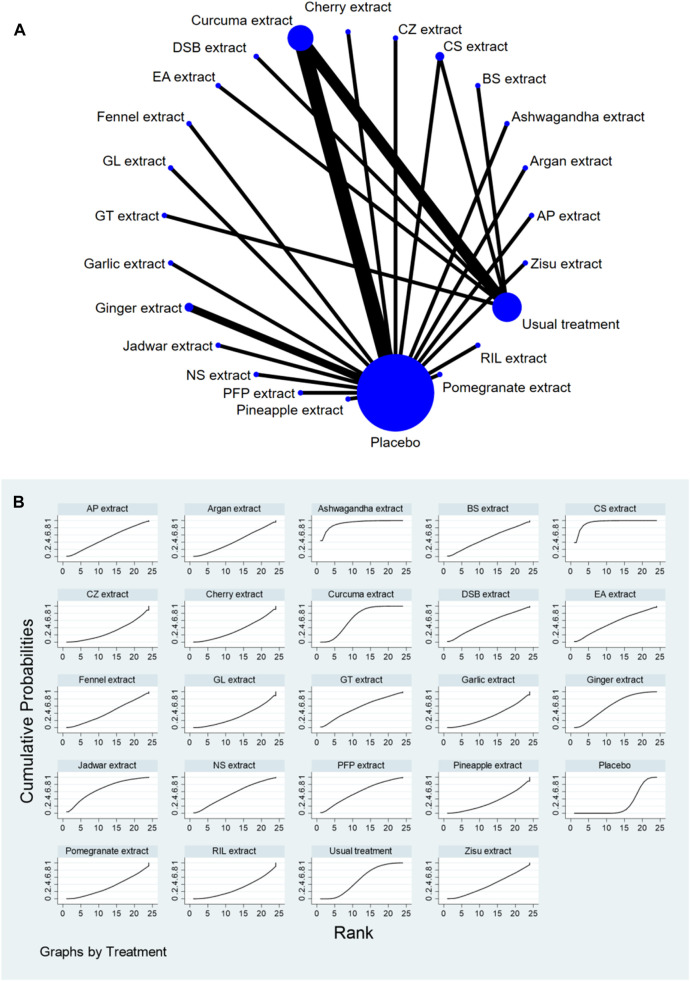
**(A)** NMA figure for Function Score. **(B)** SUCRA plot for Function Score.

The complete NMA diagrams are presented in Figures 2A, 3A, 4A.

#### 3.4.1 Knee joint pain score

The *p*-values for all indirect and direct comparisons among the studies were tested for consistency and inconsistency, with all *p*-values exceeding 0.05, indicating that the influence of inconsistency among the studies was acceptable. We conducted global inconsistency test and found that the p value was 0.63. The DIC of consistency model was 201.4, while that of inconsistency model was 208.9. ΔDIC was all >5, indicating that the consistency model was supported. Detailed information is provided in Supplementary Table S2.

The results of the network meta-analysis showed that, compared to the control group using placebo, CS extract [SMD = 6.65, 95% CI = (3.83, 9.48)] and Ashwagandha extract [SMD = 3.78, 95% CI = (0.11, 7.45)] were more effective in reducing Pain scores. Compared to the control group receiving conventional treatment, CS extract [SMD = 6.44, 95% CI = (3.58, 9.31)] was also more effective in reducing Pain scores. In the probability ranking for reducing Pain scores among different botanical and medicinal plant extracts, CS extract ranked first in SUCRA (99.2%, see Figure 2B). The comparisons between the two different interventions are presented in Table 2.

#### 3.4.2 Knee joint stiffness score

The *p*-values for all indirect and direct comparisons across the studies were tested for consistency and inconsistency, with all *p*-values exceeding 0.05, indicating that the consistency among the studies was acceptable. We conducted global inconsistency test and found that the p value was 0.57. The DIC of consistency model was 176.8, while that of inconsistency model was 184.3. ΔDIC was all >5, indicating that the consistency model was supported. Detailed information is provided in Supplementary Table S3.

The results of the network meta-analysis showed that, compared to the placebo control group, Ashwagandha extract [SMD = 4.16, 95% CI = (2.43, 5.90)], CS extract [SMD = 3.74, 95% CI = (2.58, 4.90)], Jadwar extract [SMD = 1.74, 95% CI = (0.36, 3.11)], AP extract [SMD = 1.51, 95% CI = (0.10, 2.92)], and Curcuma extract [SMD = 0.79, 95% CI = (0.12, 1.47)] were more effective in reducing Stiff scores. Compared to the control group receiving conventional treatment, Ashwagandha extract [SMD = 3.90, 95% CI = (1.88, 5.91)] and CS extract [SMD = 3.48, 95% CI = (2.32, 4.64)] were more effective in reducing Stiff scores. In the probability ranking for reducing Stiff scores among different botanical and medicinal plant extracts, Ashwagandha extract ranked first in SUCRA (98.3%, see Figure 3B). Comparisons between the two different interventions are presented in Table 4.

**TABLE 4:** League table on Stiffness Score.

Ashwagandha extract	CS extract	Jadwar extract	AP extract	NS extract	Curcuma extract	Curcuma extract	NS extract	GL extract	EA extract	Pomegranate extract	GT extract
Ashwagandha extract	0.42 (−1.67,2.51)	2.42 (0.21,4.64)	2.65 (0.42,4.89)	3.04 (0.84,5.25)	3.37 (1.51,5.23)	3.54 (1.32,5.76)	3.68 (1.71,5.65)	3.69 (1.45,5.92)	3.72 (1.28,6.17)	3.74 (1.48,6.00)	3.77 (1.30,6.25)
−0.42 (−2.51,1.67)	CS extract	2.00 (0.21,3.80)	2.23 (0.41,4.06)	2.62 (0.84,4.41)	2.95 (1.76,4.14)	3.12 (1.32,4.92)	3.26 (1.77,4.75)	3.27 (1.45,5.09)	3.31 (1.50,5.11)	3.32 (1.46,5.17)	3.36 (1.51,5.20)
−2.42 (−4.64,−0.21)	−2.00 (−3.80,−0.21)	Jadwar extract	0.23 (−1.74,2.20)	0.62 (−1.31,2.55)	0.94 (−0.59,2.48)	1.12 (−0.83,3.06)	1.25 (−0.41,2.92)	1.26 (−0.70,3.23)	1.30 (−0.91,3.51)	1.31 (−0.68,3.31)	1.35 (−0.89,3.59)
−2.65 (−4.89,−0.42)	−2.23 (−4.06,−0.41)	−0.23 (−2.20,1.74)	AP extract	0.39 (−1.57,2.35)	0.72 (−0.84,2.28)	0.89 (−1.08,2.86)	1.03 (−0.66,2.72)	1.04 (−0.95,3.03)	1.07 (−1.15,3.30)	1.09 (−0.93,3.10)	1.12 (−1.14,3.38)
−3.04 (−5.25,−0.84)	−2.62 (−4.41,−0.84)	−0.62 (−2.55,1.31)	−0.39 (−2.35,1.57)	NS extract	0.32 (−1.20,1.84)	0.50 (−1.44,2.43)	0.63 (−1.02,2.29)	0.64 (−1.31,2.60)	0.68 (−1.52,2.88)	0.69 (−1.29,2.68)	0.73 (−1.50,2.96)
−3.37 (−5.23,−1.51)	−2.95 (−4.14,−1.76)	−0.94 (−2.48,0.59)	−0.72 (−2.28,0.84)	−0.32 (−1.84,1.20)	Curcuma extract	0.17 (−1.36,1.71)	0.31 (−0.85,1.47)	0.32 (−1.24,1.88)	0.36 (−1.28,1.99)	0.37 (−1.23,1.97)	0.41 (−1.27,2.09)
−3.54 (−5.76,−1.32)	−3.12 (−4.92,−1.32)	−1.12 (−3.06,0.83)	−0.89 (−2.86,1.08)	−0.50 (−2.43,1.44)	−0.17 (−1.71,1.36)	Curcuma extract	0.14 (−1.53,1.80)	0.15 (−1.82,2.12)	0.18 (−2.02,2.39)	0.20 (−1.80,2.20)	0.23 (−2.01,2.47)
−3.68 (−5.65,−1.71)	−3.26 (−4.75,−1.77)	−1.25 (−2.92,0.41)	−1.03 (−2.72,0.66)	−0.63 (−2.29,1.02)	−0.31 (−1.47,0.85)	−0.14 (−1.80,1.53)	NS extract	0.01 (−1.68,1.70)	0.05 (−1.92,2.01)	0.06 (−1.67,1.79)	0.10 (−1.90,2.10)
−3.69 (−5.92,−1.45)	−3.27 (−5.09,−1.45)	−1.26 (−3.23,0.70)	−1.04 (−3.03,0.95)	−0.64 (−2.60,1.31)	−0.32 (−1.88,1.24)	−0.15 (−2.12,1.82)	−0.01 (−1.70,1.68)	GL extract	0.04 (−2.19,2.26)	0.05 (−1.97,2.07)	0.09 (−2.17,2.35)
−3.72 (−6.17,−1.28)	−3.31 (−5.11,−1.50)	−1.30 (−3.51,0.91)	−1.07 (−3.30,1.15)	−0.68 (−2.88,1.52)	−0.36 (−1.99,1.28)	−0.18 (−2.39,2.02)	−0.05 (−2.01,1.92)	−0.04 (−2.26,2.19)	EA extract	0.01 (−2.24,2.27)	0.05 (−1.95,2.05)
−3.74 (−6.00,−1.48)	−3.32 (−5.17,−1.46)	−1.31 (−3.31,0.68)	−1.09 (−3.10,0.93)	−0.69 (−2.68,1.29)	−0.37 (−1.97,1.23)	−0.20 (−2.20,1.80)	−0.06 (−1.79,1.67)	−0.05 (−2.07,1.97)	−0.01 (−2.27,2.24)	Pomegranate extract	0.04 (−2.25,2.32)
−3.77 (−6.25,−1.30)	−3.36 (−5.20,−1.51)	−1.35 (−3.59,0.89)	−1.12 (−3.38,1.14)	−0.73 (−2.96,1.50)	−0.41 (−2.09,1.27)	−0.23 (−2.47,2.01)	−0.10 (−2.10,1.90)	−0.09 (−2.35,2.17)	−0.05 (−2.05,1.95)	−0.04 (−2.32,2.25)	GT extract
−3.88 (−6.31,−1.45)	−3.46 (−5.24,−1.68)	−1.46 (−3.64,0.73)	−1.23 (−3.43,0.98)	−0.84 (−3.01,1.34)	−0.51 (−2.12,1.10)	−0.34 (−2.53,1.85)	−0.20 (−2.14,1.74)	−0.19 (−2.40,2.01)	−0.16 (−2.09,1.78)	−0.14 (−2.37,2.09)	−0.11 (−2.08,1.87)
−3.90 (−6.10,−1.69)	−3.48 (−5.26,−1.69)	−1.47 (−3.40,0.46)	−1.24 (−3.20,0.71)	−0.85 (−2.77,1.07)	−0.53 (−2.04,0.99)	−0.36 (−2.29,1.58)	−0.22 (−1.87,1.43)	−0.21 (−2.16,1.75)	−0.17 (−2.37,2.02)	−0.16 (−2.14,1.83)	−0.12 (−2.35,2.11)
−3.91 (−6.18,−1.63)	−3.49 (−5.36,−1.62)	−1.48 (−3.49,0.53)	−1.25 (−3.29,0.78)	−0.86 (−2.87,1.14)	−0.54 (−2.16,1.08)	−0.37 (−2.38,1.65)	−0.23 (−1.97,1.51)	−0.22 (−2.25,1.82)	−0.18 (−2.45,2.08)	−0.17 (−2.23,1.89)	−0.13 (−2.43,2.16)
−3.94 (−6.15,−1.72)	−3.52 (−5.32,−1.72)	−1.51 (−3.46,0.43)	−1.28 (−3.25,0.68)	−0.89 (−2.83,1.04)	−0.57 (−2.10,0.97)	−0.40 (−2.34,1.55)	−0.26 (−1.92,1.41)	−0.25 (−2.22,1.72)	−0.21 (−2.42,2.00)	−0.20 (−2.20,1.80)	−0.16 (−2.40,2.08)
−3.90 (−5.91,−1.88)	−3.48 (−4.64,−2.32)	−1.47 (−3.19,0.24)	−1.25 (−2.99,0.50)	−0.85 (−2.56,0.85)	−0.53 (−1.40,0.34)	−0.36 (−2.08,1.36)	−0.22 (−1.61,1.17)	−0.21 (−1.95,1.53)	−0.17 (−1.56,1.21)	−0.16 (−1.94,1.61)	−0.12 (−1.56,1.31)
−3.95 (−6.40,−1.51)	−3.53 (−5.34,−1.73)	−1.53 (−3.73,0.68)	−1.30 (−3.53,0.92)	−0.91 (−3.11,1.29)	−0.58 (−2.22,1.05)	−0.41 (−2.62,1.79)	−0.27 (−2.24,1.69)	−0.26 (−2.49,1.96)	−0.23 (−2.19,1.73)	−0.21 (−2.47,2.04)	−0.18 (−2.18,1.82)
−3.96 (−6.24,−1.69)	−3.55 (−5.42,−1.67)	−1.54 (−3.56,0.47)	−1.31 (−3.35,0.72)	−0.92 (−2.93,1.08)	−0.60 (−2.22,1.02)	−0.43 (−2.44,1.59)	−0.29 (−2.03,1.46)	−0.28 (−2.31,1.76)	−0.24 (−2.51,2.03)	−0.23 (−2.29,1.84)	−0.19 (−2.49,2.11)
−4.05 (−6.25,−1.85)	−3.63 (−5.41,−1.85)	−1.62 (−3.55,0.30)	−1.40 (−3.35,0.55)	−1.00 (−2.92,0.91)	−0.68 (−2.19,0.83)	−0.51 (−2.44,1.42)	−0.37 (−2.01,1.27)	−0.36 (−2.31,1.59)	−0.32 (−2.51,1.87)	−0.31 (−2.29,1.67)	−0.27 (−2.50,1.95)
−4.06 (−6.30,−1.81)	−3.64 (−5.48,−1.80)	−1.64 (−3.62,0.34)	−1.41 (−3.41,0.60)	−1.02 (−2.99,0.95)	−0.69 (−2.27,0.89)	−0.52 (−2.50,1.46)	−0.38 (−2.09,1.32)	−0.37 (−2.37,1.63)	−0.34 (−2.57,1.90)	−0.32 (−2.35,1.71)	−0.29 (−2.55,1.98)
−4.28 (−6.50,−2.06)	−3.86 (−5.67,−2.05)	−1.85 (−3.81,0.10)	−1.63 (−3.60,0.35)	−1.23 (−3.18,0.71)	−0.91 (−2.45,0.63)	−0.74 (−2.69,1.22)	−0.60 (−2.27,1.07)	−0.59 (−2.56,1.39)	−0.55 (−2.77,1.66)	−0.54 (−2.54,1.46)	−0.50 (−2.75,1.74)
−4.16 (−5.90,−2.43)	−3.74 (−4.90,−2.58)	−1.74 (−3.11,−0.36)	−1.51 (−2.92,−0.10)	−1.12 (−2.48,0.24)	−0.79 (−1.47,−0.12)	−0.62 (−2.00,0.76)	−0.48 (−1.42,0.45)	−0.47 (−1.88,0.94)	−0.44 (−2.16,1.29)	−0.42 (−1.87,1.03)	−0.39 (−2.15,1.38)
−4.30 (−6.50,−2.11)	−3.89 (−5.66,−2.11)	−1.88 (−3.80,0.04)	−1.65 (−3.60,0.29)	−1.26 (−3.17,0.65)	−0.94 (−2.44,0.57)	−0.76 (−2.69,1.16)	−0.63 (−2.26,1.01)	−0.62 (−2.56,1.33)	−0.58 (−2.77,1.61)	−0.57 (−2.54,1.41)	−0.53 (−2.75,1.69)

DSB extract	Argan extract	PFP extract	Zisu extract	Usual treatment	BS extract	Pineapple extract	CZ extract	Cherry extract	Fennel extract	Placebo	RIL extract
3.88 (1.45,6.31)	3.90 (1.69,6.10)	3.91 (1.63,6.18)	3.94 (1.72,6.15)	3.90 (1.88,5.91)	3.95 (1.51,6.40)	3.96 (1.69,6.24)	4.05 (1.85,6.25)	4.06 (1.81,6.30)	4.28 (2.06,6.50)	4.16 (2.43,5.90)	4.30 (2.11,6.50)
3.46 (1.68,5.24)	3.48 (1.69,5.26)	3.49 (1.62,5.36)	3.52 (1.72,5.32)	3.48 (2.32,4.64)	3.53 (1.73,5.34)	3.55 (1.67,5.42)	3.63 (1.85,5.41)	3.64 (1.80,5.48)	3.86 (2.05,5.67)	3.74 (2.58,4.90)	3.89 (2.11,5.66)
1.46 (−0.73,3.64)	1.47 (−0.46,3.40)	1.48 (−0.53,3.49)	1.51 (−0.43,3.46)	1.47 (−0.24,3.19)	1.53 (−0.68,3.73)	1.54 (−0.47,3.56)	1.62 (−0.30,3.55)	1.64 (−0.34,3.62)	1.85 (−0.10,3.81)	1.74 (0.36,3.11)	1.88 (−0.04,3.80)
1.23 (−0.98,3.43)	1.24 (−0.71,3.20)	1.25 (−0.78,3.29)	1.28 (−0.68,3.25)	1.25 (−0.50,2.99)	1.30 (−0.92,3.53)	1.31 (−0.72,3.35)	1.40 (−0.55,3.35)	1.41 (−0.60,3.41)	1.63 (−0.35,3.60)	1.51 (0.10,2.92)	1.65 (−0.29,3.60)
0.84 (−1.34,3.01)	0.85 (−1.07,2.77)	0.86 (−1.14,2.87)	0.89 (−1.04,2.83)	0.85 (−0.85,2.56)	0.91 (−1.29,3.11)	0.92 (−1.08,2.93)	1.00 (−0.91,2.92)	1.02 (−0.95,2.99)	1.23 (−0.71,3.18)	1.12 (−0.24,2.48)	1.26 (−0.65,3.17)
0.51 (−1.10,2.12)	0.53 (−0.99,2.04)	0.54 (−1.08,2.16)	0.57 (−0.97,2.10)	0.53 (−0.34,1.40)	0.58 (−1.05,2.22)	0.60 (−1.02,2.22)	0.68 (−0.83,2.19)	0.69 (−0.89,2.27)	0.91 (−0.63,2.45)	0.79 (0.12,1.47)	0.94 (−0.57,2.44)
0.34 (−1.85,2.53)	0.36 (−1.58,2.29)	0.37 (−1.65,2.38)	0.40 (−1.55,2.34)	0.36 (−1.36,2.08)	0.41 (−1.79,2.62)	0.43 (−1.59,2.44)	0.51 (−1.42,2.44)	0.52 (−1.46,2.50)	0.74 (−1.22,2.69)	0.62 (−0.76,2.00)	0.76 (−1.16,2.69)
0.20 (−1.74,2.14)	0.22 (−1.43,1.87)	0.23 (−1.51,1.97)	0.26 (−1.41,1.92)	0.22 (−1.17,1.61)	0.27 (−1.69,2.24)	0.29 (−1.46,2.03)	0.37 (−1.27,2.01)	0.38 (−1.32,2.09)	0.60 (−1.07,2.27)	0.48 (−0.45,1.42)	0.63 (−1.01,2.26)
0.19 (−2.01,2.40)	0.21 (−1.75,2.16)	0.22 (−1.82,2.25)	0.25 (−1.72,2.22)	0.21 (−1.53,1.95)	0.26 (−1.96,2.49)	0.28 (−1.76,2.31)	0.36 (−1.59,2.31)	0.37 (−1.63,2.37)	0.59 (−1.39,2.56)	0.47 (−0.94,1.88)	0.62 (−1.33,2.56)
0.16 (−1.78,2.09)	0.17 (−2.02,2.37)	0.18 (−2.08,2.45)	0.21 (−2.00,2.42)	0.17 (−1.21,1.56)	0.23 (−1.73,2.19)	0.24 (−2.03,2.51)	0.32 (−1.87,2.51)	0.34 (−1.90,2.57)	0.55 (−1.66,2.77)	0.44 (−1.29,2.16)	0.58 (−1.61,2.77)
0.14 (−2.09,2.37)	0.16 (−1.83,2.14)	0.17 (−1.89,2.23)	0.20 (−1.80,2.20)	0.16 (−1.61,1.94)	0.21 (−2.04,2.47)	0.23 (−1.84,2.29)	0.31 (−1.67,2.29)	0.32 (−1.71,2.35)	0.54 (−1.46,2.54)	0.42 (−1.03,1.87)	0.57 (−1.41,2.54)
0.11 (−1.87,2.08)	0.12 (−2.11,2.35)	0.13 (−2.16,2.43)	0.16 (−2.08,2.40)	0.12 (−1.31,1.56)	0.18 (−1.82,2.18)	0.19 (−2.11,2.49)	0.27 (−1.95,2.50)	0.29 (−1.98,2.55)	0.50 (−1.74,2.75)	0.39 (−1.38,2.15)	0.53 (−1.69,2.75)
DSB extract	0.02 (−2.16,2.19)	0.03 (−2.22,2.27)	0.06 (−2.13,2.24)	0.02 (−1.33,1.37)	0.07 (−1.86,2.01)	0.09 (−2.16,2.33)	0.17 (−2.00,2.34)	0.18 (−2.04,2.40)	0.40 (−1.79,2.59)	0.28 (−1.42,1.98)	0.42 (−1.74,2.59)
−0.02 (−2.19,2.16)	Argan extract	0.01 (−1.99,2.01)	0.04 (−1.89,1.97)	0.00 (−1.70,1.70)	0.06 (−2.14,2.25)	0.07 (−1.93,2.07)	0.15 (−1.76,2.07)	0.16 (−1.80,2.13)	0.38 (−1.56,2.32)	0.27 (−1.09,1.62)	0.41 (−1.50,2.32)
−0.03 (−2.27,2.22)	−0.01 (−2.01,1.99)	PFP extract	0.03 (−1.98,2.04)	−0.01 (−1.80,1.78)	0.05 (−2.22,2.31)	0.06 (−2.02,2.14)	0.14 (−1.85,2.14)	0.15 (−1.89,2.20)	0.37 (−1.65,2.39)	0.25 (−1.21,1.72)	0.40 (−1.59,2.39)
−0.06 (−2.24,2.13)	−0.04 (−1.97,1.89)	−0.03 (−2.04,1.98)	Zisu extract	−0.04 (−1.75,1.68)	0.02 (−2.19,2.22)	0.03 (−1.99,2.04)	0.11 (−1.82,2.04)	0.12 (−1.86,2.10)	0.34 (−1.61,2.29)	0.23 (−1.15,1.60)	0.37 (−1.55,2.29)
−0.02 (−1.37,1.33)	−0.00 (−1.70,1.70)	0.01 (−1.78,1.80)	0.04 (−1.68,1.75)	Usual treatment	0.05 (−1.33,1.44)	0.07 (−1.73,1.86)	0.15 (−1.55,1.85)	0.16 (−1.59,1.92)	0.38 (−1.34,2.10)	0.26 (−0.76,1.29)	0.41 (−1.28,2.09)
−0.07 (−2.01,1.86)	−0.06 (−2.25,2.14)	−0.05 (−2.31,2.22)	−0.02 (−2.22,2.19)	−0.05 (−1.44,1.33)	BS extract	0.01 (−2.25,2.28)	0.09 (−2.10,2.28)	0.11 (−2.13,2.34)	0.33 (−1.89,2.54)	0.21 (−1.51,1.93)	0.35 (−1.83,2.54)
−0.09 (−2.33,2.16)	−0.07 (−2.07,1.93)	−0.06 (−2.14,2.02)	−0.03 (−2.04,1.99)	−0.07 (−1.86,1.73)	−0.01 (−2.28,2.25)	Pineapple extract	0.08 (−1.92,2.08)	0.09 (−1.96,2.14)	0.31 (−1.71,2.33)	0.20 (−1.28,1.67)	0.34 (−1.65,2.33)
−0.17 (−2.34,2.00)	−0.15 (−2.07,1.76)	−0.14 (−2.14,1.85)	−0.11 (−2.04,1.82)	−0.15 (−1.85,1.55)	−0.09 (−2.28,2.10)	−0.08 (−2.08,1.92)	CZ extract	0.01 (−1.95,1.97)	0.23 (−1.70,2.17)	0.11 (−1.24,1.46)	0.26 (−1.65,2.16)
−0.18 (−2.40,2.04)	−0.16 (−2.13,1.80)	−0.15 (−2.20,1.89)	−0.12 (−2.10,1.86)	−0.16 (−1.92,1.59)	−0.11 (−2.34,2.13)	−0.09 (−2.14,1.96)	−0.01 (−1.97,1.95)	Cherry extract	0.22 (−1.77,2.21)	0.10 (−1.32,1.53)	0.25 (−1.71,2.20)
−0.40 (−2.59,1.79)	−0.38 (−2.32,1.56)	−0.37 (−2.39,1.65)	−0.34 (−2.29,1.61)	−0.38 (−2.10,1.34)	−0.33 (−2.54,1.89)	−0.31 (−2.33,1.71)	−0.23 (−2.17,1.70)	−0.22 (−2.21,1.77)	Fennel extract	−0.12 (−1.50,1.27)	0.03 (−1.90,1.95)
−0.28 (−1.98,1.42)	−0.27 (−1.62,1.09)	−0.25 (−1.72,1.21)	−0.23 (−1.60,1.15)	−0.26 (−1.29,0.76)	−0.21 (−1.93,1.51)	−0.20 (−1.67,1.28)	−0.11 (−1.46,1.24)	−0.10 (−1.53,1.32)	0.12 (−1.27,1.50)	Placebo	0.14 (−1.20,1.48)
−0.42 (−2.59,1.74)	−0.41 (−2.32,1.50)	−0.40 (−2.39,1.59)	−0.37 (−2.29,1.55)	−0.41 (−2.09,1.28)	−0.35 (−2.54,1.83)	−0.34 (−2.33,1.65)	−0.26 (−2.16,1.65)	−0.25 (−2.20,1.71)	−0.03 (−1.95,1.90)	−0.14 (−1.48,1.20)	RIL extract

#### 3.4.3 Knee joint function score

The *p*-values for all indirect and direct comparisons across the studies were tested for consistency and inconsistency, with all *p*-values exceeding 0.05, indicating that the consistency among the studies was acceptable. We conducted global inconsistency test and found that the p value was 0.43. The DIC of consistency model was 223.7, while that of inconsistency model was 230.1. ΔDIC was all >5, indicating that the consistency model was supported. Detailed information is provided in Supplementary Table S4.

The results of the network meta-analysis showed that, compared to the placebo control group, CS extract [SMD = 4.28, 95% CI = (2.08, 6.49)], Ashwagandha extract [SMD = 4.32, 95% CI = (1.34, 7.31)], and Curcuma extract [SMD = 1.78, 95% CI = (0.44, 3.11)] were more effective in reducing Function scores. Compared to the control group receiving conventional treatment, CS extract [SMD = 2.94, 95% CI = (0.73, 5.15)] was more effective in reducing Function scores. In the probability ranking for reducing Function scores among different botanical and medicinal plant extracts, CS extract ranked first in SUCRA (94.6%, see Figure 4B). Comparisons between the two different interventions are presented in Table 5.

**TABLE 5:** League table on Function Score.

CS extract	Ashwagandha extract	Jadwar extract	Curcuma extract	NS extract	PFP extract	GT extract	Usual treatment	NS extract	DSB extracts	EA extract	AP extract
CS extract	−0.04 (−3.75,3.67)	2.20 (−1.35,5.75)	2.51 (0.24,4.77)	2.67 (−0.27,5.62)	2.72 (−0.88,6.33)	2.81 (−0.76,6.38)	2.94 (0.73,5.15)	2.96 (−0.58,6.50)	2.95 (−0.59,6.48)	2.96 (−0.59,6.52)	3.16 (−0.39,6.71)
0.04 (−3.67,3.75)	Ashwagandha extract	2.24 (−1.84,6.32)	2.55 (−0.72,5.82)	2.71 (−0.85,6.28)	2.76 (−1.36,6.89)	2.85 (−1.65,7.36)	2.98 (−0.54,6.51)	3.00 (−1.07,7.07)	2.99 (−1.49,7.47)	3.01 (−1.48,7.50)	3.20 (−0.88,7.28)
−2.20 (−5.75,1.35)	−2.24 (−6.32,1.84)	Jadwar extract	0.30 (−2.78,3.39)	0.47 (−2.93,3.87)	0.52 (−3.46,4.50)	0.61 (−3.76,4.99)	0.74 (−2.62,4.09)	0.76 (−3.17,4.68)	0.75 (−3.60,5.09)	0.76 (−3.59,5.12)	0.96 (−2.98,4.89)
−2.51 (−4.77,−0.24)	−2.55 (−5.82,0.72)	−0.30 (−3.39,2.78)	Curcuma extract	0.17 (−2.20,2.53)	0.22 (−2.93,3.37)	0.31 (−2.87,3.49)	0.43 (−1.06,1.93)	0.45 (−2.62,3.53)	0.44 (−2.70,3.58)	0.46 (−2.70,3.62)	0.65 (−2.44,3.74)
−2.67 (−5.62,0.27)	−2.71 (−6.28,0.85)	−0.47 (−3.87,2.93)	−0.17 (−2.53,2.20)	NS extract	0.05 (−3.40,3.51)	0.14 (−3.76,4.04)	0.27 (−2.44,2.97)	0.29 (−3.10,3.68)	0.27 (−3.59,4.14)	0.29 (−3.59,4.17)	0.49 (−2.91,3.89)
−2.72 (−6.33,0.88)	−2.76 (−6.89,1.36)	−0.52 (−4.50,3.46)	−0.22 (−3.37,2.93)	−0.05 (−3.51,3.40)	PFP extract	0.09 (−4.33,4.51)	0.22 (−3.20,3.63)	0.24 (−3.74,4.21)	0.22 (−4.17,4.62)	0.24 (−4.16,4.65)	0.44 (−3.55,4.42)
−2.81 (−6.38,0.76)	−2.85 (−7.36,1.65)	−0.61 (−4.99,3.76)	−0.31 (−3.49,2.87)	−0.14 (−4.04,3.76)	−0.09 (−4.51,4.33)	GT extract	0.13 (−2.68,2.93)	0.15 (−4.22,4.51)	0.13 (−3.81,4.07)	0.15 (−3.80,4.10)	0.35 (−4.03,4.72)
−2.94 (−5.15,−0.73)	−2.98 (−6.51,0.54)	−0.74 (−4.09,2.62)	−0.43 (−1.93,1.06)	−0.27 (−2.97,2.44)	−0.22 (−3.63,3.20)	−0.13 (−2.93,2.68)	Usual treatment	0.02 (−3.33,3.37)	0.01 (−2.76,2.77)	0.03 (−2.76,2.81)	0.22 (−3.14,3.58)
−2.96 (−6.50,0.58)	−3.00 (−7.07,1.07)	−0.76 (−4.68,3.17)	−0.45 (−3.53,2.62)	−0.29 (−3.68,3.10)	−0.24 (−4.21,3.74)	−0.15 (−4.51,4.22)	−0.02 (−3.37,3.33)	NS extract	−0.01 (−4.35,4.33)	0.01 (−4.35,4.36)	0.20 (−3.73,4.13)
−2.95 (−6.48,0.59)	−2.99 (−7.47,1.49)	−0.75 (−5.09,3.60)	−0.44 (−3.58,2.70)	−0.27 (−4.14,3.59)	−0.22 (−4.62,4.17)	−0.13 (−4.07,3.81)	−0.01 (−2.77,2.76)	0.01 (−4.33,4.35)	DSB extracts	0.02 (−3.90,3.94)	0.21 (−4.14,4.56)
−2.96 (−6.52,0.59)	−3.01 (−7.50,1.48)	−0.76 (−5.12,3.59)	−0.46 (−3.62,2.70)	−0.29 (−4.17,3.59)	−0.24 (−4.65,4.16)	−0.15 (−4.10,3.80)	−0.03 (−2.81,2.76)	−0.01 (−4.36,4.35)	−0.02 (−3.94,3.90)	EA extract	0.19 (−4.17,4.56)
−3.16 (−6.71,0.39)	−3.20 (−7.28,0.88)	−0.96 (−4.89,2.98)	−0.65 (−3.74,2.44)	−0.49 (−3.89,2.91)	−0.44 (−4.42,3.55)	−0.35 (−4.72,4.03)	−0.22 (−3.58,3.14)	−0.20 (−4.13,3.73)	−0.21 (−4.56,4.14)	−0.19 (−4.56,4.17)	AP extract
−3.14 (−6.69,0.41)	−3.18 (−7.67,1.31)	−0.93 (−5.29,3.42)	−0.63 (−3.79,2.53)	−0.46 (−4.34,3.42)	−0.41 (−4.82,3.99)	−0.32 (−4.27,3.63)	−0.20 (−2.98,2.58)	−0.18 (−4.53,4.17)	−0.19 (−4.11,3.73)	−0.17 (−4.10,3.76)	0.02 (−4.34,4.38)
−3.57 (−7.11,−0.03)	−3.61 (−7.68,0.46)	−1.37 (−5.29,2.55)	−1.07 (−4.14,2.01)	−0.90 (−4.29,2.49)	−0.85 (−4.82,3.13)	−0.76 (−5.13,3.61)	−0.63 (−3.98,2.71)	−0.61 (−4.53,3.30)	−0.62 (−4.96,3.71)	−0.61 (−4.96,3.74)	−0.41 (−4.34,3.51)
−3.59 (−7.14,−0.04)	−3.63 (−7.71,0.45)	−1.39 (−5.33,2.54)	−1.09 (−4.17,2.00)	−0.92 (−4.32,2.48)	−0.87 (−4.85,3.11)	−0.78 (−5.16,3.60)	−0.65 (−4.01,2.70)	−0.63 (−4.56,3.29)	−0.65 (−4.99,3.70)	−0.63 (−4.99,3.73)	−0.43 (−4.37,3.50)
−3.71 (−7.26,−0.17)	−3.76 (−7.83,0.32)	−1.51 (−5.44,2.42)	−1.21 (−4.29,1.87)	−1.04 (−4.44,2.35)	−0.99 (−4.97,2.99)	−0.90 (−5.27,3.47)	−0.78 (−4.13,2.58)	−0.76 (−4.68,3.17)	−0.77 (−5.11,3.58)	−0.75 (−5.11,3.61)	−0.56 (−4.49,3.38)
−3.97 (−7.54,−0.39)	−4.01 (−8.11,0.09)	−1.77 (−5.72,2.19)	−1.46 (−4.57,1.65)	−1.29 (−4.72,2.13)	−1.24 (−5.25,2.76)	−1.15 (−5.55,3.24)	−1.03 (−4.41,2.35)	−1.01 (−4.96,2.94)	−1.02 (−5.39,3.35)	−1.00 (−5.38,3.38)	−0.81 (−4.77,3.15)
−4.08 (−7.64,−0.51)	−4.12 (−8.21,−0.03)	−1.87 (−5.82,2.07)	−1.57 (−4.67,1.53)	−1.40 (−4.82,2.01)	−1.35 (−5.35,2.64)	−1.26 (−5.65,3.12)	−1.14 (−4.51,2.24)	−1.12 (−5.06,2.82)	−1.13 (−5.49,3.23)	−1.11 (−5.48,3.26)	−0.92 (−4.87,3.03)
−4.12 (−7.66,−0.57)	−4.16 (−8.23,−0.08)	−1.92 (−5.84,2.01)	−1.61 (−4.69,1.47)	−1.44 (−4.84,1.95)	−1.39 (−5.37,2.58)	−1.30 (−5.68,3.07)	−1.18 (−4.53,2.17)	−1.16 (−5.08,2.76)	−1.17 (−5.51,3.17)	−1.15 (−5.51,3.20)	−0.96 (−4.89,2.97)
−4.21 (−7.77,−0.65)	−4.25 (−8.34,−0.17)	−2.01 (−5.95,1.93)	−1.71 (−4.80,1.39)	−1.54 (−4.94,1.87)	−1.49 (−5.48,2.50)	−1.40 (−5.78,2.98)	−1.27 (−4.64,2.09)	−1.25 (−5.18,2.68)	−1.26 (−5.62,3.09)	−1.25 (−5.61,3.12)	−1.05 (−5.00,2.89)
−4.21 (−7.79,−0.62)	−4.25 (−8.36,−0.14)	−2.00 (−5.97,1.96)	−1.70 (−4.82,1.43)	−1.53 (−4.97,1.90)	−1.48 (−5.50,2.53)	−1.39 (−5.80,3.01)	−1.27 (−4.66,2.13)	−1.25 (−5.20,2.71)	−1.26 (−5.63,3.12)	−1.24 (−5.63,3.15)	−1.05 (−5.01,2.92)
−4.22 (−7.75,−0.68)	−4.26 (−8.32,−0.19)	−2.01 (−5.93,1.90)	−1.71 (−4.78,1.36)	−1.54 (−4.92,1.84)	−1.49 (−5.46,2.47)	−1.40 (−5.76,2.96)	−1.28 (−4.61,2.06)	−1.26 (−5.17,2.65)	−1.27 (−5.60,3.06)	−1.25 (−5.60,3.09)	−1.06 (−4.98,2.86)
−4.28 (−7.81,−0.74)	−4.32 (−8.39,−0.25)	−2.08 (−6.00,1.84)	−1.77 (−4.84,1.30)	−1.61 (−4.99,1.78)	−1.55 (−5.53,2.42)	−1.46 (−5.83,2.90)	−1.34 (−4.68,2.00)	−1.32 (−5.23,2.60)	−1.33 (−5.67,3.01)	−1.31 (−5.66,3.03)	−1.12 (−5.04,2.81)
−4.28 (−6.49,−2.08)	−4.32 (−7.31,−1.34)	−2.08 (−4.86,0.70)	−1.78 (−3.11,−0.44)	−1.61 (−3.56,0.34)	−1.56 (−4.41,1.29)	−1.47 (−4.85,1.91)	−1.34 (−3.22,0.53)	−1.32 (−4.09,1.45)	−1.34 (−4.68,2.01)	−1.32 (−4.67,2.04)	−1.12 (−3.91,1.66)

BS extract	Argan extract	Fennel extract	Zisu extract	Pomegranate extract	Cherry extract	Garlic extract	GL extract	Pineapple extract	RIL extract	CZ extract	Placebo
3.14 (−0.41,6.69)	3.57 (0.03,7.11)	3.59 (0.04,7.14)	3.71 (0.17,7.26)	3.97 (0.39,7.54)	4.08 (0.51,7.64)	4.12 (0.57,7.66)	4.21 (0.65,7.77)	4.21 (0.62,7.79)	4.22 (0.68,7.75)	4.28 (0.74,7.81)	4.28 (2.08,6.49)
3.18 (−1.31,7.67)	3.61 (−0.46,7.68)	3.63 (−0.45,7.71)	3.76 (−0.32,7.83)	4.01 (−0.09,8.11)	4.12 (0.03,8.21)	4.16 (0.08,8.23)	4.25 (0.17,8.34)	4.25 (0.14,8.36)	4.26 (0.19,8.32)	4.32 (0.25,8.39)	4.32 (1.34,7.31)
0.93 (−3.42,5.29)	1.37 (−2.55,5.29)	1.39 (−2.54,5.33)	1.51 (−2.42,5.44)	1.77 (−2.19,5.72)	1.87 (−2.07,5.82)	1.92 (−2.01,5.84)	2.01 (−1.93,5.95)	2.00 (−1.96,5.97)	2.01 (−1.90,5.93)	2.08 (−1.84,6.00)	2.08 (−0.70,4.86)
0.63 (−2.53,3.79)	1.07 (−2.01,4.14)	1.09 (−2.00,4.17)	1.21 (−1.87,4.29)	1.46 (−1.65,4.57)	1.57 (−1.53,4.67)	1.61 (−1.47,4.69)	1.71 (−1.39,4.80)	1.70 (−1.43,4.82)	1.71 (−1.36,4.78)	1.77 (−1.30,4.84)	1.78 (0.44,3.11)
0.46 (−3.42,4.34)	0.90 (−2.49,4.29)	0.92 (−2.48,4.32)	1.04 (−2.35,4.44)	1.29 (−2.13,4.72)	1.40 (−2.01,4.82)	1.44 (−1.95,4.84)	1.54 (−1.87,4.94)	1.53 (−1.90,4.97)	1.54 (−1.84,4.92)	1.61 (−1.78,4.99)	1.61 (−0.34,3.56)
0.41 (−3.99,4.82)	0.85 (−3.13,4.82)	0.87 (−3.11,4.85)	0.99 (−2.99,4.97)	1.24 (−2.76,5.25)	1.35 (−2.64,5.35)	1.39 (−2.58,5.37)	1.49 (−2.50,5.48)	1.48 (−2.53,5.50)	1.49 (−2.47,5.46)	1.55 (−2.42,5.53)	1.56 (−1.29,4.41)
0.32 (−3.63,4.27)	0.76 (−3.61,5.13)	0.78 (−3.60,5.16)	0.90 (−3.47,5.27)	1.15 (−3.24,5.55)	1.26 (−3.12,5.65)	1.30 (−3.07,5.68)	1.40 (−2.98,5.78)	1.39 (−3.01,5.80)	1.40 (−2.96,5.76)	1.46 (−2.90,5.83)	1.47 (−1.91,4.85)
0.20 (−2.58,2.98)	0.63 (−2.71,3.98)	0.65 (−2.70,4.01)	0.78 (−2.58,4.13)	1.03 (−2.35,4.41)	1.14 (−2.24,4.51)	1.18 (−2.17,4.53)	1.27 (−2.09,4.64)	1.27 (−2.13,4.66)	1.28 (−2.06,4.61)	1.34 (−2.00,4.68)	1.34 (−0.53,3.22)
0.18 (−4.17,4.53)	0.61 (−3.30,4.53)	0.63 (−3.29,4.56)	0.76 (−3.17,4.68)	1.01 (−2.94,4.96)	1.12 (−2.82,5.06)	1.16 (−2.76,5.08)	1.25 (−2.68,5.18)	1.25 (−2.71,5.20)	1.26 (−2.65,5.17)	1.32 (−2.60,5.23)	1.32 (−1.45,4.09)
0.19 (−3.73,4.11)	0.62 (−3.71,4.96)	0.65 (−3.70,4.99)	0.77 (−3.58,5.11)	1.02 (−3.35,5.39)	1.13 (−3.23,5.49)	1.17 (−3.17,5.51)	1.26 (−3.09,5.62)	1.26 (−3.12,5.63)	1.27 (−3.06,5.60)	1.33 (−3.01,5.67)	1.34 (−2.01,4.68)
0.17 (−3.76,4.10)	0.61 (−3.74,4.96)	0.63 (−3.73,4.99)	0.75 (−3.61,5.11)	1.00 (−3.38,5.38)	1.11 (−3.26,5.48)	1.15 (−3.20,5.51)	1.25 (−3.12,5.61)	1.24 (−3.15,5.63)	1.25 (−3.09,5.60)	1.31 (−3.03,5.66)	1.32 (−2.04,4.67)
−0.02 (−4.38,4.34)	0.41 (−3.51,4.34)	0.43 (−3.50,4.37)	0.56 (−3.38,4.49)	0.81 (−3.15,4.77)	0.92 (−3.03,4.87)	0.96 (−2.97,4.89)	1.05 (−2.89,5.00)	1.05 (−2.92,5.01)	1.06 (−2.86,4.98)	1.12 (−2.81,5.04)	1.12 (−1.66,3.91)
BS extract	0.44 (−3.91,4.79)	0.46 (−3.90,4.82)	0.58 (−3.78,4.93)	0.83 (−3.55,5.21)	0.94 (−3.43,5.31)	0.98 (−3.37,5.34)	1.08 (−3.29,5.44)	1.07 (−3.32,5.46)	1.08 (−3.26,5.42)	1.14 (−3.20,5.49)	1.15 (−2.21,4.50)
−0.44 (−4.79,3.91)	Argan extract	0.02 (−3.90,3.95)	0.14 (−3.78,4.06)	0.40 (−3.55,4.34)	0.50 (−3.43,4.44)	0.55 (−3.37,4.47)	0.64 (−3.29,4.57)	0.63 (−3.32,4.59)	0.64 (−3.26,4.55)	0.71 (−3.21,4.62)	0.71 (−2.06,3.48)
−0.46 (−4.82,3.90)	−0.02 (−3.95,3.90)	Fennel extract	0.12 (−3.81,4.05)	0.37 (−3.58,4.33)	0.48 (−3.46,4.43)	0.52 (−3.41,4.46)	0.62 (−3.32,4.56)	0.61 (−3.35,4.58)	0.62 (−3.30,4.54)	0.68 (−3.24,4.61)	0.69 (−2.09,3.47)
−0.58 (−4.93,3.78)	−0.14 (−4.06,3.78)	−0.12 (−4.05,3.81)	Zisu extract	0.25 (−3.70,4.20)	0.36 (−3.58,4.31)	0.40 (−3.52,4.33)	0.50 (−3.44,4.43)	0.49 (−3.47,4.45)	0.50 (−3.41,4.42)	0.56 (−3.36,4.48)	0.57 (−2.21,3.35)
−0.83 (−5.21,3.55)	−0.40 (−4.34,3.55)	−0.37 (−4.33,3.58)	−0.25 (−4.20,3.70)	Pomegranate extract	0.11 (−3.86,4.08)	0.15 (−3.80,4.10)	0.24 (−3.72,4.21)	0.24 (−3.75,4.22)	0.25 (−3.69,4.19)	0.31 (−3.63,4.25)	0.32 (−2.50,3.13)
−0.94 (−5.31,3.43)	−0.50 (−4.44,3.43)	−0.48 (−4.43,3.46)	−0.36 (−4.31,3.58)	−0.11 (−4.08,3.86)	Cherry extract	0.04 (−3.90,3.98)	0.14 (−3.82,4.09)	0.13 (−3.85,4.11)	0.14 (−3.79,4.07)	0.20 (−3.73,4.14)	0.21 (−2.59,3.01)
−0.98 (−5.34,3.37)	−0.55 (−4.47,3.37)	−0.52 (−4.46,3.41)	−0.40 (−4.33,3.52)	−0.15 (−4.10,3.80)	−0.04 (−3.98,3.90)	Garlic extract	0.09 (−3.84,4.03)	0.09 (−3.87,4.05)	0.10 (−3.81,4.01)	0.16 (−3.76,4.08)	0.17 (−2.61,2.94)
−1.08 (−5.44,3.29)	−0.64 (−4.57,3.29)	−0.62 (−4.56,3.32)	−0.50 (−4.43,3.44)	−0.24 (−4.21,3.72)	−0.14 (−4.09,3.82)	−0.09 (−4.03,3.84)	GL extract	−0.01 (−3.98,3.97)	0.00 (−3.92,3.93)	0.07 (−3.86,3.99)	0.07 (−2.72,2.86)
−1.07 (−5.46,3.32)	−0.63 (−4.59,3.32)	−0.61 (−4.58,3.35)	−0.49 (−4.45,3.47)	−0.24 (−4.22,3.75)	−0.13 (−4.11,3.85)	−0.09 (−4.05,3.87)	0.01 (−3.97,3.98)	Pineapple extract	0.01 (−3.94,3.96)	0.07 (−3.88,4.02)	0.08 (−2.75,2.90)
−1.08 (−5.42,3.26)	−0.64 (−4.55,3.26)	−0.62 (−4.54,3.30)	−0.50 (−4.42,3.41)	−0.25 (−4.19,3.69)	−0.14 (−4.07,3.79)	−0.10 (−4.01,3.81)	−0.00 (−3.93,3.92)	−0.01 (−3.96,3.94)	RIL extract	0.06 (−3.84,3.97)	0.07 (−2.69,2.83)
−1.14 (−5.49,3.20)	−0.71 (−4.62,3.21)	−0.68 (−4.61,3.24)	−0.56 (−4.48,3.36)	−0.31 (−4.25,3.63)	−0.20 (−4.14,3.73)	−0.16 (−4.08,3.76)	−0.07 (−3.99,3.86)	−0.07 (−4.02,3.88)	−0.06 (−3.97,3.84)	CZ extract	0.00 (−2.76,2.77)
−1.15 (−4.50,2.21)	−0.71 (−3.48,2.06)	−0.69 (−3.47,2.09)	−0.57 (−3.35,2.21)	−0.32 (−3.13,2.50)	−0.21 (−3.01,2.59)	−0.17 (−2.94,2.61)	−0.07 (−2.86,2.72)	−0.08 (−2.90,2.75)	−0.07 (−2.83,2.69)	−0.00 (−2.77,2.76)	Placebo

### 3.5 Adverse events and safety outcomes

In terms of safety, the included botanical and plant-based preparations were generally well tolerated. Across trials, most adverse events were mild and gastrointestinal in nature. For example, ginger extract was associated with dyspepsia or heartburn in a small number of participants but without any treatment-related serious adverse events (Altman and Marcussen, 2001; Wigler et al., 2003). Bromelain mainly caused transient gastrointestinal upset without severe consequences (Brien et al., 2006). In a trial of curcumin co-administered with diclofenac, adverse events such as renal impairment and allergic swelling occurred only in the diclofenac group, whereas the combination group reported only a single case of hair loss (Pinsornsak and Niempoog, 2012). More recently, perilla extract was associated with only mild and unlikely drug-related adverse events and no laboratory abnormalities (Kim et al., 2023). Overall, no trial reported life-threatening or irreversible harms, and in several studies the safety profile compared favorably with conventional pharmacological comparators.

### 3.6 Publication bias test

**FIGURE 5:**
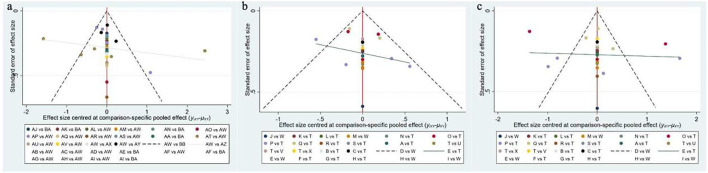
Funnel plot on publication bias. **(a)** Pain Score; **(b)** Stiffness Score; **(c)** Function score.

We constructed independent funnel plots for all outcome measures to assess potential publication bias. Visual inspection of the funnel plots revealed no significant evidence of publication bias. Detailed information is presented in Figure 5.

## 4 Discussion

This study compared the efficacy of various botanical and medicinal plant extract interventions in treating patients with KOA. A total of 36 studies, comprising 26 different botanical and medicinal plant extracts and 3,285 patients diagnosed with KOA, were included. Our analysis indicated that CS extract was the most effective botanical and medicinal plant extract for reducing knee pain scores and functional impairment scores, while Ashwagandha extract demonstrated better efficacy in reducing knee stiffness scores. Overall, we consider CS extract to be the most suitable intervention for improving KOA symptoms. Given that there are only two studies with high homogeneity but small sample size for Cucumis sativus, the first result should be regarded as an exploratory signal and needs to be verified by a large sample multi-center RCT with independent teams.

From the results, it is evident that CS extract demonstrated the most significant therapeutic effect in alleviating pain scores among patients. Pain is the predominant symptom of KOA, typically characterized by mild to severe aching discomfort within the joint. In more severe instances, patients may experience tearing or pinprick sensations that persist even during rest and are often influenced by changes in weather conditions. The pathophysiology of KOA pain primarily arises from degenerative wear of joint cartilage, leading to an uneven articular surface that causes friction and impact during movement (Gelber, 2024). Furthermore, synovial inflammation due to cartilage damage releases inflammatory mediators that stimulate nerve endings, further contributing to joint pain (Tudorachi et al., 2021). CS extract contains a rare imino sugar amino acid, idoBR1 (Olajide et al., 2022), which has emerged as a novel anti-inflammatory molecule with excellent oral bioavailability and *in vivo* stability. Imino sugar amino acids are extremely rare in nature and exhibit no significant functional or structural similarity to current drugs for the treatment of osteoarthritis. Studies have shown that idoBR1 can effectively inhibit lipopolysaccharide (LPS)-induced production of the pro-inflammatory cytokine TNF-α in human serum and THP-1 cells, where elevated TNF-α levels are a key factor in degenerative changes (Nash et al., 2023). Additionally, idoBR1 was found to dose-dependently reduce LPS-induced TNF-α levels and significantly suppress multiple inflammatory markers, including interleukin-6 (IL-6), nitric oxide (NO), and transcription factor NF-κB, demonstrating its comprehensive anti-inflammatory potential(Nash et al., 2020; Olajide et al., 2022). The meta-analysis included two RCTs on CS extract, which showed that it exhibited the best therapeutic effects in improving pain. Additionally, its SUCRA ranking was second for alleviating knee stiffness. While Curcuma extract and BS extract have been promising in recent studies for KOA treatment (Liu et al., 2018; Yu et al., 2020), our study found CS extract to be more effective than both. In addition, other extracts are also considered to have certain clinical value, Ashwagandha extract can significantly reduce the release of pro-inflammatory cytokines such as IL-1β and TNF-α in synovial fluid monocytes by inhibiting the activation of the NF-κB signaling pathway, thereby alleviating the inflammatory response of knee joint synovium. It can also cooperate with chondroitin sulfate to upregulate the synthesis of glycosaminoglycans (GAGs) in articular cartilage and reduce the excessive production of nitric oxide (NO), thereby maintaining the homeostasis of cartilage matrix. The main active metabolite of Curcuma, curcumin, can block the NF-κB and MAPK cascades, inhibit the expression of cartilage-degrading enzymes such as MMP-3 and MMP-13, and thereby delay the destruction of articular cartilage. At the same time, curcumin can reduce oxidative stress damage in the joint cavity by enhancing the activity of superoxide dismutase (SOD) and reducing lipid peroxidation products, further improving the microenvironment of knee osteoarthritis.

Patients with KOA often experience joint stiffness, reduced fluidity of movement, and even locking sensations during activity, with stiffness being particularly prominent in the morning. These symptoms are often accompanied by joint swelling, pain, and restricted range of motion. The pathophysiology is complex, involving inflammation-induced synovial hyperplasia, congestion, and edema, along with structural changes such as capsular thickening and contracture, which significantly impair normal joint movement (Gelber, 2024). Inflammation may also lead to the accumulation of synovial fluid in the joint cavity, further exacerbating restrictions in joint mobility (Tudorachi et al., 2021). Prolonged inflammation can result in tension, spasms, and even atrophy of the surrounding muscles, further worsening joint stiffness and creating a vicious cycle. Ashwagandha (Indian ginseng) is a traditional Ayurvedic botanical drug. A previous study reported that Ashwagandha extract significantly reduced NO release in cartilage samples from osteoarthritis patients, demonstrating its anti-inflammatory effects (Sumantran et al., 2008). Clinically, it has been shown that after 4 and 8 weeks of Ashwagandha extract administration in KOA patients, patients showed statistically significant reductions in all efficacy variables in a dose-dependent manner (Ramakanth et al., 2016). However, studies on this botanical medicine remain limited, and its mechanisms and potential benefits in alleviating joint stiffness require further investigation.

Similarly, CS extract demonstrated the most effective treatment in reducing knee functional limitation scores. The degenerative wear and destruction of articular cartilage, reactive proliferation of subchondral bone forming osteophytes, and inflammatory reactions in the joint synovium result in abnormal joint structure, thereby affecting normal joint function (Gelber, 2024). The superior anti-inflammatory properties of CS extract exhibit better therapeutic outcomes in clinical practice compared to both placebo and chondroitin sulfate.

In conclusion, this study has a certain clinical significance. CS extract has a significant effect on relieving pain, stiffness and functional improvement of knee osteoarthritis, and it is a good natural extract for the treatment of KOA.

## 5 Strengths and limitations

Our study included 36 studies and 3,285 patients, representing a relatively large sample size. We summarized the efficacy of various botanical and medicinal plant extracts for treating KOA patients from databases, with each study comparing botanical and medicinal plant extracts to placebo or conventional treatment interventions, providing updated and more comprehensive evidence-based recommendations.

However, our study shares some common limitations with the studies on which it is based. Although we made every effort to control for heterogeneity when including the original studies, heterogeneity between studies was inevitable (e.g., differences in the proportion of studies conducted in various regions and gender disparities among participants). Although 36 studies were included in our analysis, the number of studies for each type of botanical and medicinal plant extract was relatively small, with most botanical and medicinal plant extracts supported by only 1–2 RCTs. In addition, certain studies had limited evaluation criteria, and their results should be interpreted with caution. This highlights the need for further research to expand the evidence base. Most of the clinical trials included in this systematic review were conducted by Asian research teams, and the subjects were mainly from Asian populations. Due to differences in genetic background, lifestyle, dietary habits, and physical constitution, the efficacy and safety of botanical interventions observed in Asian populations may not be directly extrapolated to other racial or regional populations. Therefore, the applicability of the existing evidence to non-Asian populations remains uncertain. Currently, nearly all plant extracts/formulations in the field are only compared with placebos or conventional treatments, resulting in a severe lack of head-to-head randomized controlled trials (RCTs). To reduce uncertainties from indirect comparisons, future studies should prioritize multi-arm designs or platform trials that directly compare 2-3 of the most promising botanical extracts. Standardizing dosage regimens, treatment durations, and outcome measures will enhance the robustness of network meta-analysis results.

## 6 Conclusion

Based on our findings, we recommend CS extract for patients seeking to alleviate knee pain and improve joint function, and Ashwagandha extract for those aiming to reduce knee stiffness. Overall, CS extract is the most recommended botanical and medicinal plant extract for osteoarthritis patients aiming to improve overall knee joint symptoms.

This study has been registered with PROSPERO, registration number: CRD42024617459.

## Data availability statement

The original contributions presented in the study are included in the article/Supplementary Material, further inquiries can be directed to the corresponding authors.
