# Supplementary material for: Efficacy of botanical extracts for knee osteoarthritis: a network meta-analysis of randomized controlled trials
Source: Front Pharmacol. 2025 Oct 7;16:1619589. doi: 10.3389/fphar.2025.1619589 (PMC12537895; doi:10.3389/fphar.2025.1619589)
Supplement: Supplementary file 2 [file Supplementaryfile6.doc]

| **Element** | **Description** |
| --- | --- |
| **Population (P)** | Adults diagnosed with knee osteoarthritis (KOA) according to clinical or radiological criteria, across multiple global regions (Asia, Europe, Americas, Africa). |
| **Intervention (I)** | Single herbal or medicinal plant extracts, including Zingiber officinale (ginger), Ananas comosus (pineapple, bromelain), Boswellia serrata, Passiflora edulis (passion fruit peel), Derris scandens, Curcuma longa (turmeric), Sesamum indicum (sesame), Prunus cerasus (tart cherry), Olea europaea (olive leaf), Punica granatum (pomegranate), Elaeagnus angustifolia, Withania somnifera (ashwagandha), Argania spinosa (argan), Camellia sinensis (green tea), Psidium guajava (guava leaf), Momordica charantia (bitter melon), Cucumis sativus (cucumber), Allium sativum (garlic), Andrographis paniculata, Foeniculum vulgare (fennel), Chrysanthemum zawadskii, Rubus idaeus (raspberry leaf), Nigella sativa (black seed), Perilla frutescens (zisu), Delphinium denudatum (jadwar), Drimia maritima (squill oxymel). |
| **Comparator (C)** | Placebo or conventional treatment (e.g., NSAIDs, paracetamol, standard care). |
| **Outcomes (O)** | Primary: WOMAC (pain, stiffness, function); Secondary: VAS, KOOS, SF-36, LPFI, JOA. |
| **Timing (T)** | Duration of interventions ranged from 4 weeks to 6 months. |
| **Study Design (S)** | Randomized Controlled Trials (RCTs), double-blind or single-blind, parallel or crossover designs. |
