# Supplementary material for: Efficacy of botanical extracts for knee osteoarthritis: a network meta-analysis of randomized controlled trials
Source: Front Pharmacol. 2025 Oct 7;16:1619589. doi: 10.3389/fphar.2025.1619589 (PMC12537895; doi:10.3389/fphar.2025.1619589)
Supplement: Supplementary file 5 [file Supplementaryfile5.doc]

**PUBMED**
(

"ginger extract"[tiab] OR "Zingiber officinale"[tiab] OR "ginger rhizome"[tiab] OR

"ginger root"[tiab] OR "rhizoma zingiberis"[tiab] OR "zingiberis rhizoma"[tiab] OR

"zingiberis rhizoma praeparatum"[tiab] OR "zingiberis siccatum rhizoma"[tiab] OR

"rhizoma zingiberis recens"[tiab] OR "pineapple"[tiab] OR "Boswellia serrata"[tiab] OR

"passion fruit peel"[tiab] OR "Derris scandens"[tiab] OR "Curcuma"[tiab] OR

"turmeric"[tiab] OR "sesame"[tiab] OR "cherry"[tiab] OR "olive leaf"[tiab] OR

"pomegranate"[tiab] OR "Elaeagnus angustifolia"[tiab] OR "ashwagandha"[tiab] OR

"argan"[tiab] OR "green tea"[tiab] OR "guava leaf"[tiab] OR

"Momordica charantia"[tiab] OR "Cucumis sativus"[tiab] OR "garlic"[tiab] OR

"Andrographis paniculata"[tiab] OR "fennel"[tiab] OR

"Chrysanthemum zawadskii"[tiab] OR "Rubus idaeus leaf"[tiab] OR

"Nigella sativa"[tiab] OR "zisu"[tiab] OR "jadwar"[tiab] OR "squill oxymel"[tiab]

)

AND

(

"knee osteoarthritis"[tiab] OR "knee osteoarthritides"[tiab] OR

"osteoarthritis of knee"[tiab] OR "osteoarthritis of the knee"[tiab] OR

"osteoarthritis"[tiab] OR "knee joint"[tiab]

)

**Web Of Science**

TS=(

("ginger extract" OR "Zingiber officinale" OR "ginger rhizome" OR "ginger root" OR

"rhizoma zingiberis" OR "zingiberis rhizoma" OR "zingiberis rhizoma praeparatum" OR

"zingiberis siccatum rhizoma" OR "rhizoma zingiberis recens" OR pineapple OR

"Boswellia serrata" OR "passion fruit peel" OR "Derris scandens" OR Curcuma OR

turmeric OR sesame OR cherry OR "olive leaf" OR pomegranate OR

"Elaeagnus angustifolia" OR ashwagandha OR argan OR "green tea" OR

"guava leaf" OR "Momordica charantia" OR "Cucumis sativus" OR garlic OR

"Andrographis paniculata" OR fennel OR "Chrysanthemum zawadskii" OR

"Rubus idaeus leaf" OR "Nigella sativa" OR zisu OR jadwar OR "squill oxymel")

AND

("knee osteoarthritis" OR "knee osteoarthritides" OR "osteoarthritis of knee" OR

"osteoarthritis of the knee" OR osteoarthritis OR "knee joint")

)

**Embase**

(ginger extract or zingiber officinale or ginger rhizome or ginger root or

rhizoma zingiberis or zingiberis rhizoma or zingiberis rhizoma praeparatum or

zingiberis siccatum rhizoma or rhizoma zingiberis recens or pineapple or

boswellia serrata or passion fruit peel or derris scandens or curcuma or

turmeric or sesame or cherry or olive leaf or pomegranate or

elaeagnus angustifolia or ashwagandha or argan or green tea or

guava leaf or momordica charantia or cucumis sativus or garlic or

andrographis paniculata or fennel or chrysanthemum zawadskii or

rubus idaeus leaf or nigella sativa or zisu or jadwar or squill oxymel).ti,ab

AND

(knee osteoarthritis or knee osteoarthritides or osteoarthritis of knee or

osteoarthritis of the knee or osteoarthritis or knee joint).ti,ab

**Cochrane library**

ti,ab,kw(

("ginger extract" OR "Zingiber officinale" OR "ginger rhizome" OR "ginger root" OR

"rhizoma zingiberis" OR "zingiberis rhizoma" OR "zingiberis rhizoma praeparatum" OR

"zingiberis siccatum rhizoma" OR "rhizoma zingiberis recens" OR pineapple OR

"Boswellia serrata" OR "passion fruit peel" OR "Derris scandens" OR curcuma OR

turmeric OR sesame OR cherry OR "olive leaf" OR pomegranate OR

"Elaeagnus angustifolia" OR ashwagandha OR argan OR "green tea" OR

"guava leaf" OR "Momordica charantia" OR "Cucumis sativus" OR garlic OR

"Andrographis paniculata" OR fennel OR "Chrysanthemum zawadskii" OR

"Rubus idaeus leaf" OR "Nigella sativa" OR zisu OR jadwar OR "squill oxymel")

AND

("knee osteoarthritis" OR "knee osteoarthritides" OR "osteoarthritis of knee" OR

"osteoarthritis of the knee" OR osteoarthritis OR "knee joint")

)
